# High diversity and novel host associations of bat haemosporidian parasites in Cambodia: insights from a nationwide bat survey and multilocus analyses

**DOI:** 10.1186/s13071-026-07732-3

**Published:** 2026-10-08

**Authors:** Juliane Schaer, Oskar Werb, Lena Ch´ng, Dolyce Hong Wen Low, Sophie Alison Borthwick, Neil Furey, Liesbeth Frias, Awatsaya Pimsai, Raksmey Yim, Kimashalen Chor, Dany Cheang, Gavin J. D. Smith, Alan Hitch, Ian H. Mendenhall

**Affiliations:** 1 Institute of Biology, Humboldt University, Berlin, Germany; 2 Department of Biology, Muni University, Arua, Uganda; 3 Department of Biological Sciences, Macquarie University, North Ryde, NSW Australia; 4 Duke-NUS Medical School, Singapore, Singapore; 5 Fauna & Flora, Phnom Penh, Cambodia; 6 Harrison Institute, Sevenoaks, Kent UK; 7 Princess Maha Chakri Sirindhorn Natural History Museum, Prince of Songkla University, Hat Yai, Songkhla, Thailand; 8 Forestry Administration, Phnom Penh, Cambodia; 9 Museum of Fish and Wildlife Biology, Department of Wildlife, Fisheries and Conservation Biology, University of California, Davis, CA USA 95616; 10 School of Analytics and Computational Sciences, Harrisburg University of Science and Technology, Harrisburg, PA USA 17101

**Keywords:** Chiroptera, Bats, Haemosporida, Malaria, Hepatocystis, Phylogeny, Southeast Asia

## Abstract

**Background:**

Haemosporidian (“malaria”) parasites are a diverse group of vector-borne apicomplexan parasites that infect a wide range of vertebrate hosts, including humans. Bats harbour a remarkable diversity of haemosporidians and are considered important hosts in the evolution of mammalian haemosporidian lineages, yet parasite diversity, host associations, and evolutionary relationships remain poorly understood, particularly in biodiversity-rich Southeast Asia.

**Methods:**

We conducted a nationwide molecular survey of 1180 bats representing 34 species across Cambodia and combined extensive sampling with multilocus phylogenetic and ecological analyses to investigate haemosporidian diversity, host associations, evolutionary relationships, and ecological correlates of infection.

**Results:**

Haemosporidian infections were detected in 12.9% of the sampled bats and comprised diverse *Polychromophilus* and *Hepatocystis* taxa, including several novel host associations. Notably, *Hepatocystis* was detected in Emballonuridae for the first time and occurred across multiple bat families. We substantially expand sampling of a previously described *Scotophilus*-associated *Polychromophilus* lineage and demonstrate its remarkable genetic conservation across geographic regions. In contrast, *Hepatocystis* parasites exhibited substantial genetic diversity across multiple host taxa. Multi-gene analyses expanded phylogenetic representation of bat-associated *Hepatocystis* and revealed close relationships between some bat- and squirrel-associated lineages. These data further suggest considerable diversity among Asian *Hepatocystis* parasites and phylogenetic patterns consistent with diversification across Asian host groups. Ecological analyses indicated that the infection varied more among host species and sampling sites than with measured host traits or landscape attributes and revealed pronounced seasonal differences in infection probability together with lower infection rate in more densely populated landscapes.

**Conclusions:**

Our findings expand current knowledge of bat haemosporidian diversity, host associations, and evolutionary relationships in Southeast Asia. Novel host associations, including the first record of *Hepatocystis* in Emballonuridae, highlight previously undocumented parasite diversity in regional bat communities. Infection was structured principally by host species and by conditions at individual roosting sites rather than by broader landscape composition, indicating that fine-scale roost ecology and vector biology are the more promising targets for understanding transmission in bats. Together, these findings contribute to a broader understanding of diversification and host associations in mammalian malaria parasites.

**Graphical Abstract:**

**Figure:**
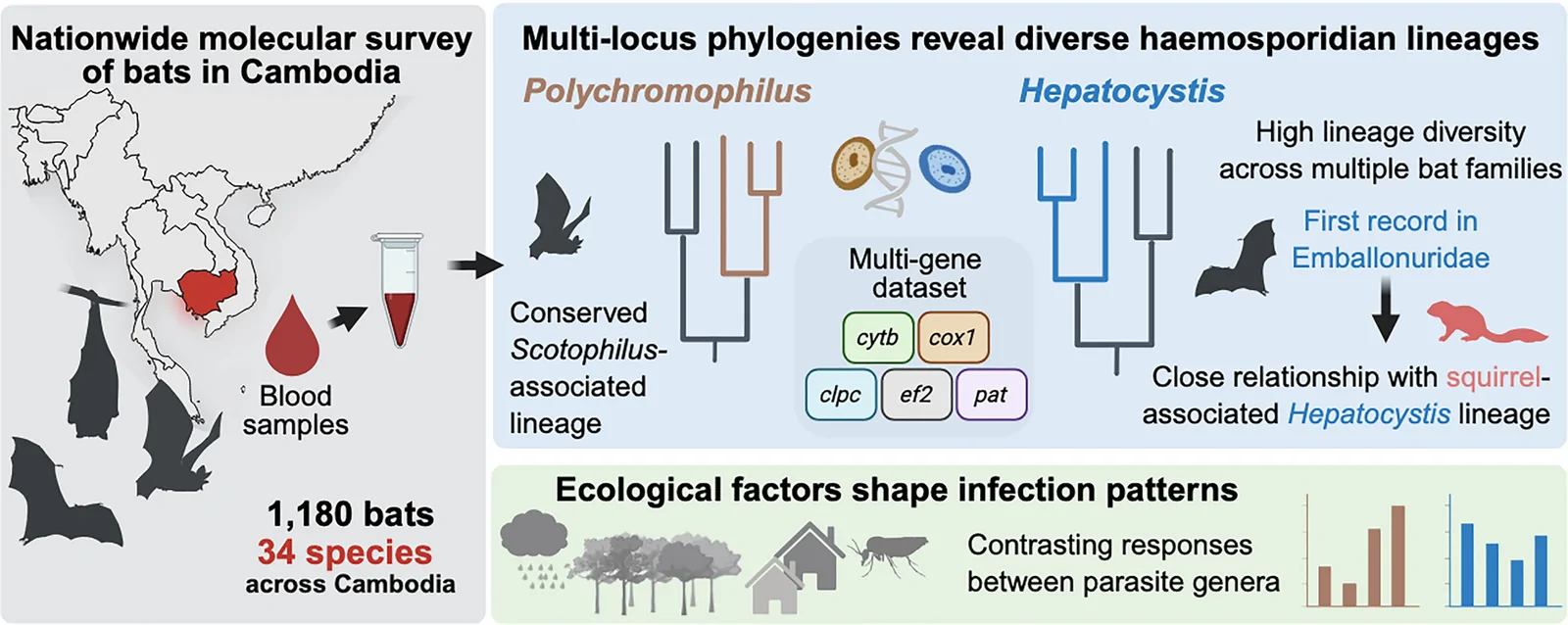

**Supplementary Information:**

The online version contains supplementary material available at https://doi.org/10.1186/s13071-026-07732-3.

## Background

Malaria, a significant vector-borne disease, is caused by eukaryotic protozoan parasites of the genus *Plasmodium* (Order Haemosporida). While *Plasmodium* species infecting humans and rodents have been studied extensively, they represent only a small fraction of the remarkable diversity of haemosporidian parasites that infect mammals and other vertebrates [1, 2].

Bats (Order Chiroptera) comprise more than 20% of the extant mammalian species [3] and are of particular importance as hosts for haemosporidian parasites. They harbour parasites from at least seven genera, collectively representing approximately one-third of all described haemosporidian species infecting mammals [2]. These parasites infect bats across Europe, Africa, Asia, Australia, and the Americas [4]. Despite this diversity and broad distribution, major gaps remain in our understanding of bat haemosporidian diversity, host associations, evolutionary relationships, and transmission dynamics. Investigating haemosporidian parasites in bats therefore provides an important opportunity to better understand the evolution of malarial parasites in mammals [2]. Because haemosporidian parasites rely on blood-feeding arthropod vectors for transmission, variation in host ecology, roosting behaviour, and environmental conditions can strongly influence parasite distribution and infection dynamics.

Bats appear to have played a key role in the evolutionary history of Haemosporida, with parasite lineages undergoing substantial divergence following host switches into bat hosts [5]. However, phylogenetic relationships within several haemosporidian groups remain unresolved, including within the genus *Hepatocystis* and among bat-restricted genera such as *Nycteria* and *Polychromophilus*. These uncertainties are largely driven by limited taxon sampling and the uneven availability of molecular data across host groups and geographical regions [4].

Southeast Asia, one of the most biodiverse regions globally, harbours nearly 27% of all bat species [6]. Despite this exceptional diversity, bat haemosporidian parasites remain poorly characterized in the region, limiting our understanding of their diversity, host associations, evolutionary relationships, and ecological drivers of infection.

Here, we investigate haemosporidian infections in bats from Cambodia using a nationwide dataset comprising 1180 bats representing 34 species sampled across 233 sites, combined with multilocus phylogenetic analyses and ecological modelling. This approach allows us to (i) characterize parasite diversity and novel host associations, (ii) resolve evolutionary relationships within key parasite genera, and (iii) evaluate ecological correlates that may influence parasite occurrence. By integrating large-scale parasite screening, multilocus phylogenetics, and ecological analyses, we aim to improve understanding of haemosporidian diversity, host associations, and ecological drivers of infection in bats while providing new insights into the evolutionary relationships of mammalian malaria parasites.

## Methods

### Field methods

**Fig. 1:**
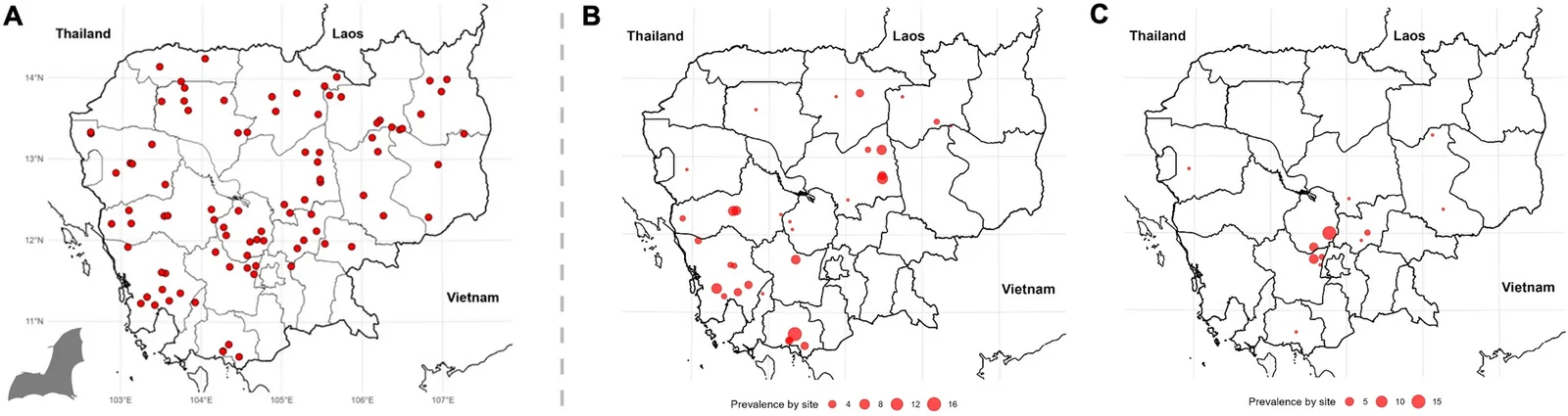
Study system and infection prevalence of haemosporidian infections in Cambodian bats. **A** Geographic distribution of sampling sites across Cambodia where bats were screened for haemosporidian parasites. **B** Prevalence of *Hepatocystis* infections in *Cynopterus* spp., *Hipposideros* spp., and *Taphozous* bats (n = 583 individuals, 81 sites). **C** Prevalence of *Polychromophilus* infections in *Scotophilus* bats (*n* = 90 individuals, 25 sites). Prevalence is shown as the proportion of infected individuals per sampling site

As part of a nationwide CamBatRat (CBR) bio-surveillance initiative, 2461 bats were sampled across 233 sites in Cambodia between December 2016 and February 2020 (Fig. 1A). The original purpose of the initiative was to investigate bat–habitat relationships and develop probabilistic models to support the spatial targeting of zoonotic disease surveillance. The sampling design comprised both probabilistically and opportunistically selected sites. Probabilistically selected sites were randomly generated in ArcGIS Desktop (version 10.5.1) and stratified by habitat type using official 2009 landcover data from the Forestry Administration of the Cambodian Ministry of Agriculture, Forestry and Fisheries. Bats were surveyed at each site for a single night using mist nets, with harp traps additionally deployed when site assessment deemed them useful.

Samples collected through this programme were subsequently used for the investigation of haemosporidian parasites reported here. Of the 2461 bats sampled during the CBR initiative, 1180 individuals representing 34 species were selected for retrospective haemosporidian screening, with the aim of representing the bat species captured during the survey as comprehensively as possible while accounting for the availability of suitable archived blood samples.

Standard morphological measurements, sex, and age data were recorded, and species were identified following Kruskop [7] and Francis [8]. Field identifications were validated using photographs of diagnostic characters and genetic barcoding. Individuals belonging to the *Hipposideros larvatus sensu lato*, a geographically widespread species complex that comprises several morphologically similar forms whose taxonomic limits remain unresolved [9, 10], were treated as *H*. cf. *grandis*.

Blood was collected by puncturing the propatagial (cephalic) or uropatagial (saphenous) vein using a 27G needle, allowing blood to pool above the vein. The pooled blood was then collected and transferred into a 2-mL screw-cap tube. Immediately after collection, the blood was diluted 1:10 with 1X PBS, based on the calculated volume. The sample was allowed to clot at room temperature for 30 min, followed by centrifugation at 4000 rpm for 10 min. The serum was then transferred to a fresh 2 mL screw-cap tube for further processing. No blood smears were collected during the initial sampling, as the decision to screen this sample set for haemosporidian parasites was made retrospectively.

### DNA extraction, parasite screening and sequencing

A total of 1180 bats were screened for infections with haemosporidian parasites (Table 1). Genomic DNA was extracted from blood samples using the QIAGEN (69504, 69506) DNeasy Blood and Tissue kit following the manufacturer’s protocol with the following adjustments. We transferred a maximum of 50 µl of blood pellet to a 1.5 mL tube. After adding 20 µl Proteinase K, we adjusted the volume with PBS to 220 µL. The sample was incubated with Buffer AL at 56 °C for 10 min. DNA was eluted in a final volume of 200 µl. All samples were initially screened for the presence of haemosporidian parasite DNA with a PCR amplifying a ~ 450 bp fragment of the mitochondrial cytochrome *b* (*cytb*) gene. In selected bat taxa, an apicoplast marker (*clpC*) was additionally amplified to account for mitochondrial genome rearrangements previously reported in some bat haemosporidians that may impair amplification with standard mitochondrial primers [11]. All PCRs were carried out using the *Taq* PCR Master Mix Kit (QIAGEN^®^, 201445) in total PCR volumes of 25 µl (12.5 µl of Taq MasterMix, 1.25 µl of each primer, 2 µl of DNA template).

**Table 1:** Prevalence of haemosporidian infections in bats from Cambodia based on verification of haemosporidian DNA sequences

Bat family^§^	Bat species	Ind. infected/Ind. screened	Prevalence in %	Parasite taxon detected
Emballonuridae	*Saccolaimus saccolaimus*	0/1	0	–
	Taphozous longimanus*	0/10	0	–
	*Taphozous melanopogon*	15/83	18.1	*Hepatocystis*
Hipposideridae	*Hipposideros armiger*	0/28	0	–
	*Hipposideros diadema*	1/2	50.0	*Polychromophilus*
	*Hipposideros galeritus*	1/2	50.0	*Hepatocystis*
	*Hipposideros* cf.* grandis*	26/114	22.8	*Hepatocystis*
Megadermatidae	Lyroderma sinense**	0/3	0	–
	*Megaderma spasma*	0/13	0	–
Molossidae	*Mops plicatus*	0/288	0	–
Pteropodidae	*Cynopterus brachyotis*	43/102	42.2	*Hepatocystis*
	*Cynopterus horsfieldii*	0/1	0	–
	*Cynopterus sphinx*	26/241	10.8	*Hepatocystis*
	*Eonycteris spelaea*	1/13	7.7	*Hepatocystis*
	*Macroglossus sobrinus*	0/8	0	–
	*Megaerops niphanae*	2/28	7.1	*Hepatocystis*
	*Rousettus amplexicaudatus*	0/1	0	–
	*Rousettus leschenaultii*	0/54	0	–
Rhinolophidae	*Rhinolophus shameli*	0/57	0	–
	*Rhinolophus acuminatus*	0/8	0	–
	*Rhinolophus affinis*	0/2	0	–
	*Rhinolophus chaseni*	0/5	0	–
	*Rhinolophus thailandensis*	0/1	0	–
	*Rhinolophus malayanus*	0/8	0	–
	*Rhinolophus microglobosus*	0/2	0	–
Vespertilionidae	*Hesperoptenus blanfordi*	0/1	0	–
	*Hesperoptenus tickelli*	0/5	0	–
	*Hypsugo dolichodon*	0/1	0	–
	*Myotis ater*	0/2	0	–
	*Myotis muricola*	0/2	0	–
	*Myotis rosseti*	1/2	50.0	*Polychromophilus*
	Alionoctula paterculus***	0/2	0	–
	*Scotophilus heathii*	1/25	4.0	*Polychromophilus*
	*Scotophilus kuhlii*	35/65	53.8	*Polychromophilus*
	Total	152/1180	12.88	

^*^We recovered one cox1 *Hepatocystis* sequence, but were not successful in repeated attempts to amplify *cytb, clpC, ef2, pat* for the sample and concluded that the *cox1* sequence might represent a contamination and therefore excluded it from subsequent analyses

^**^[66]

^***^[67]

^§^Although haemosporidians have not yet been documented from Molossidae, both Megadermatidae and Rhinolophidae are recognized hosts of haemosporidian parasites (Megadermatidae: *Nycteria* [34, 36]; Rhinolophidae: *Nycteria* [13, 68–71], *Polychromophilus* [72, 73], *Plasmodium* [44], *Hepatocystis* [36]). Prevalence is based on PCR-positive individuals. Eleven PCR-positive individuals from which no parasite sequence could be generated are counted as uninfected in the ecological analyses, so prevalence in Table 1 differs slightly from that in the ecological sample

Positive samples were further characterized using additional mitochondrial (*cytb*, *cox1*), apicoplast (*clpC*), and nuclear (*ef2*, *pat*) loci, following Martinsen et al. [12], and Schaer et al. [13, 14]. These markers were selected to account for different evolutionary rates among the parasites’ three genomes and to improve phylogenetic resolution. PCR conditions and primer details are provided in Additional file 1: Table S1 and S2. Purified PCR products were eluted in 30 µL Buffer NE and Sanger-sequenced from both ends at least once to verify the accuracy of the sequences. All sequences generated in this study have been deposited in GenBank under accession numbers PX831502–PX831690.

### Phylogenetic and haplotype analyses

Sequences were edited, aligned with MAFFT [15, 16], and manually inspected in Geneious Prime v. 2024.0.1 (https://www.geneious.com). The phylogenetic relationships were inferred using Bayesian analyses in MrBayes 3.2.7 with the default priors [17], via the CIPRES Science Gateway Web Portal V3.3. (https://doi.org/10.1109/GCE.2010.5676129). Data were divided into partitions according to genes and codon positions of the protein-coding genes were defined. Different DNA substitution models and partition schemes were tested in PartitionFinder v2 [18] and the best partition schemes and models were used for each analysis (see Additional file 1: Table S3). Bayesian support was inferred for the nodes in MrBayes by sampling every 1,000 generations. Convergence was assessed using standard criteria (PSRF 1.00–1.02; ESS > 1000; average standard deviation of the posterior probability below 0.01). Following convergence, the first 25% of the samples were discarded as burn-in.

Separate analyses were conducted for *Polychromophilus* and *Hepatocystis*, with dataset composition chosen to maximize taxon coverage. For *Polychromophilus* parasites, a concatenated mitochondrial dataset (*cytb* + *cox1*; 2,541 nt, no gaps) of 76 sequences was analyzed. The dataset comprised 35.7% missing data due to the limited availability of *cox1* sequences for some of the reference taxa. Bayesian inference analysis was carried out for 10 million generations; despite the missing data, resolution and robustness in phylogenetic analysis were achieved. The avian-infecting haemosporidian taxon *Leucocytozoon* was selected as the outgroup.

As most published reference sequences of *Hepatocystis* parasites constitute partial *cytb* sequences, we first evaluated the phylogenetic relationships of *Hepatocystis* parasites based on a 927 nt alignment of *cytb* (without gaps) to include as many representative *Hepatocystis* taxa from different mammalian host groups as possible. The final analysis comprised 124 taxa and only 3% missing data. Bayesian inference analysis was carried out for 25 million generations and *Plasmodium bubalis*, a water buffalo haemosporidian, was selected as the outgroup. To evaluate lineage stability, we further conducted individual gene sequence analyses for *cox1* (915 nt alignment of 73 sequences)*, clpC* (504 nt alignment of 73 sequences) and *pat* (831 nt alignment of 53 sequences), including representative sequences of all available *Hepatocystis* taxa*.* The details for each analysis are provided in Additional file 1: Table S3. All resulting phylogenetic trees were visualized in *FigTree* v1.4.4 (https://github.com/rambaut/figtree/). Concatenated multilocus analyses were not performed for *Hepatocystis* because molecular data beyond *cytb* were largely unavailable for most reference taxa, resulting in extensive missing data and reduced phylogenetic resolution. Independent analyses of individual loci therefore maximized taxon representation while minimizing missing data.

Haplotype networks across five loci for different *Polychromophilus* and *Hepatocystis* groups were constructed in PopART v.1.7 [19] using median-joining methods to assess the genetic diversity within parasite lineages. Mixed infections were identified based on ambiguous nucleotide positions and excluded from network analyses. Sequence alignments were trimmed to obtain uniform sequence lengths.

### Ecological analysis

Infection status for the ecological analyses was defined as sequence-confirmed infection. Eleven individuals across six host species were PCR-positive but yielded no interpretable parasite sequence; these were treated as uninfected, since parasite identity could not be established. Analyses were restricted to the 663 individuals belonging to host genera in which infection was confirmed, of which 589 (88.8%) had complete covariate records.

#### Spatial data preparation

All spatial data were prepared in R 4.6.1 [20] using the *terra* (version 1.9–34; [21]), the *sf* (version 1.1–2; [22, 23]), and the *landscapemetrics* (version 2.2.1; [24]) packages. Capture localities were recorded with a handheld GPS receiver and projected to UTM zone 48 N for all distance- and area-based calculations. All buffered covariates share a single circular footprint centred on the site, so that composition, configuration, and population covariates describe the same area.

#### Land cover

Land cover was obtained from the ESA CCI Land Cover product [v2, 2016–2021] [25] at approximately 300 m resolution and reclassified into six analysis classes (agriculture, forest, mosaic, urban, water, bare), of which agriculture, forest, and mosaic were treated as focal habitat. This layer is contemporaneous with the sampling period (December 2016–February 2020) and is the source of all land cover covariates entering the models. It should be distinguished from the 2009 national land cover classification, which was used solely to stratify the random selection of sampling sites prior to fieldwork and does not enter the models.

Land cover class at the site was extracted as the value of the raster cell containing the site. Compositional proportions were calculated as the proportion of buffer cells in each class; non-focal cells were excluded and the three focal proportions renormalized to sum to one, so that each proportion describes the composition of modelled habitat within the buffer rather than of the buffer as a whole. The excluded fraction was retained per site and was negligible at most sites (median 0.00, mean 0.01), exceeding 5% at 5 of 95 sites at the 2 km radius.

#### Landscape configuration and human population density

Edge density (ED) is the total length of boundary between land cover patches within a defined landscape divided by the area of that landscape, expressed in m ha⁻^1^. It is a standard measure of landscape configuration: for a given amount of habitat, higher ED indicates a more subdivided and interdigitated arrangement, with a greater proportion of the landscape close to a habitat boundary. ED was calculated at the landscape level with landscapemetrics::sample_lsm() using a circular sampling plot. Non-focal classes were masked to NA prior to computation, so that boundaries between focal habitat and excluded cover types (for example, open water) do not contribute edge; ED therefore indexes fragmentation of focal habitat rather than total landscape heterogeneity. Because ED is sensitive to raster grain, the resolution given above should be considered when comparing these values with published estimates. The observed values ranged from 0 to 25.6 m ha⁻^1^.

Human population density was obtained from WorldPop (www.worldpop.org) (khm_pd_2020_1km, individuals km⁻^2^, 1 km resolution, 2020) [26], projected to the analysis CRS, and summarized as the mean cell value within the same buffer (range 0.2 to 604 individuals km⁻^2^). Edge density and human population density were used as proxies for habitat fragmentation and urbanization, respectively.

#### Scale

The landscape covariates were extracted within circular buffers of 2 km and 5 km radius around each site. A 500 m radius was also considered but discarded: at the 300 m grain of the land cover layer such a buffer contains approximately eight cells, too few to characterize landscape configuration. The 2 km radius is reported as the primary analysis; estimates at 5 km are given in Additional file 1: Table S4.

#### Antecedent rainfall

Rainfall was derived from CHIRPS v2.0 monthly precipitation (Climate Hazards Center, University of California Santa Barbara) [27], at 0.05° (approximately 5.5 km) resolution, cropped to Cambodia. For each survey event, rainfall was summed over the three complete calendar months immediately preceding the month of sampling; the month of sampling itself was excluded because only part of it precedes the capture. Because the CHIRPS grain exceeds the buffer radius, precipitation was extracted at the site rather than averaged over the buffer. The resulting covariate (range 27–1520 mm) therefore varies with both site location and sampling date. Season was defined as wet (May–October) or dry (November–April) according to the month of capture, following the southwest monsoon.

#### Host trait covariates

Host species were assigned to foraging guilds following the flight-morphology and foraging-ecology classification of McKenzie et al. [28], which distinguishes five strategies: forest-interior insectivores (I), edge and gap insectivores foraging in partially cluttered space (II), open-space insectivores (III), frugivores and nectarivores foraging locally in partially cluttered space between canopies (IV), and frugivores and nectarivores foraging over large areas in open airspace (V). Species in our analyzed sample fell into strategies II, III, and IV. Roost type was recorded as the primary roost substrate used by each species (caves, including rock crevices and subterranean sites; or foliage, including tree cavities and hollows), and typical roost size as an ordered category (< 10, <100, <1,000 individuals) reflecting colony sizes observed in Cambodia. Roost size assignments are approximate and were compiled from field observation and published accounts rather than systematic colony counts.

Body condition was calculated for each individual as the ratio of body mass (g) to forearm length (mm) and standardized to zero mean and unit standard deviation before entering the model. This index is not adjusted for interspecific differences in body size, so between-species variation in the index is absorbed by the host species varying intercept and the coefficient should be interpreted as a within-species association.

### Statistical analysis

#### Model structure

We modelled haemosporidian infection status as a Bernoulli response with a logit link, fitted in a Bayesian framework. For individual bat *i*,

$$y{\text{i }}\sim {\text{ Bernoulli}}\left( {p{\mathrm{i}}} \right){\text{ logit}}\left( {p{\mathrm{i}}} \right) \, = \alpha + {\mathbf{X}}{\mathrm{i}}{{\boldsymbol{\upbeta}}} + u{\mathrm{s}}\left[ {\mathrm{i}} \right] \, + v{\mathrm{k}}\left[ {\mathrm{i}} \right]u{\text{s }}\sim {\text{ Normal}}\left( {0,\sigma {\mathrm{species}}} \right),s = { 1 } \ldots S$$

$$v{\text{k }}\sim {\text{ Normal}}\left( {0,\sigma {\mathrm{site}}} \right),k = { 1 } \ldots K$$

where *y*i is infection status determined by sequencing, **X**i is the vector of covariates for individual *i*, **β** the corresponding population-level coefficients, and *u*s[i] and *v*k[i] are varying intercepts for the host species *s* and sampling site *k* of individual *i*. The two grouping factors are crossed rather than nested: species were sampled at multiple sites and sites yielded multiple species.

Host species and sampling site were treated as group-level (random) effects because they are exchangeable units drawn from larger populations of species and locations, because the number of levels is sufficient to identify a variance (*S* = 10, *K* = 84), and because partial pooling is required here: four host species had no confirmed infections and as fixed effects their coefficients would be unidentified.

All remaining covariates were treated as population-level (fixed) effects with treatment contrasts. These variables—host sex, reproductive status, roost type, typical roost size, feeding guild, foraging guild, season, and land cover class at the site—take two or three levels each, and those levels are fixed rather than sampled from a wider population. Estimating a variance from two or three deviations would leave the hyperparameter largely prior-determined and would shrink the level contrasts by an amount not supported by the data.

Reference levels were [female] for sex, [non-reproductive] for reproductive status, [cave] for roost type, [< 10] for roost size, [insectivore] for feeding guild, [II] for foraging guild, [dry] for season, and [forest] for land cover class. All continuous covariates were centred and scaled to unit standard deviation, so their coefficients are interpretable per standard deviation.

#### Level in variation for host traits

Because these four traits are invariant within a host species, their effects are estimated from contrasts among the 10 species sampled rather than among the 589 individuals screened, and they compete with the species varying intercept for the same between-species variation. We report these coefficients with their full uncertainty and interpret them accordingly. No pair of these traits partitions the host species identically, so their effects are separable in principle, although the precision available from 10 species is low.

#### Covariate set and model comparison

We fit a single integrated model containing all covariates as the basis for inference, for two reasons. First, partitioning covariates among models prevents any coefficient from being interpreted as an adjusted effect. Second, because *Polychromophilus* was detected only in *Scotophilus* and *Hepatocystis* only in *Cynopterus*, *Hipposideros*, and *Taphozous*, splitting by parasite genus also splits by host genus: within the resulting subsets, roost type and feeding guild partition the host species identically, and within the *Scotophilus* subset three of the four host traits take a single level and are not estimable. Neither problem arises when all host genera are analyzed together.

The missing values occurred only in individual-level host variables (reproductive status, *n* = 45; body condition, *n* = 38; sex, *n* = 8) and not in any site- or event-level covariate. Infection prevalence among excluded individuals (21.6%) was comparable to that among retained individuals (22.2%), and exclusions were distributed proportionally across host genera and sampling designs (Additional file 1: Table S5).

#### Priors

We used weakly regularizing priors centred at zero: Normal(0, 1.5) for the intercept, Normal(0, 1) for all population-level coefficients, and a half-Normal(0, 1) for both group-level standard deviations. Because continuous covariates are standardized, a Normal(0, 1) prior on the logit scale admits odds ratios of roughly 0.14 to 7.4 per standard deviation at two prior standard deviations, which is permissive relative to effects plausible in this system while excluding the implausibly large values a flat prior would allow. Prior predictive simulation confirmed that the priors imply a distribution of population prevalence spanning plausible values without concentrating near zero or one.

Posterior estimates were compared under three prior specifications: the regularizing priors above, the brms default priors, and an alternative specification with priors centred away from zero for body condition, edge density, and human population density (Additional file 1: Table S6).

#### Estimation and diagnostics

Models were fitted with brms (2.23.0) [29] as an interface to Stan (2.32.7) [30], in R (4.6.1) [20]. We ran four chains of 4000 iterations each, discarding the first 2000 as warm-up, with adapt_delta set to 0.99 and maximum tree depth to 12, giving 8000 post-warm-up draws per model.

Convergence and sampler behaviour were assessed by split-R̂, bulk and tail effective sample size, the number of divergent transitions, the number of iterations saturating the maximum tree depth, and the energy Bayesian fraction of missing information. Across all models R̂ did not exceed 1.00, the minimum bulk effective sample size was 2097 and the minimum tail effective sample size 3502, and there were no divergent transitions and no iterations saturating maximum tree depth (Additional file 1: Table S7). Model fit was assessed by graphical posterior predictive checks, overall and grouped by host species and land cover class (Additional file 1: Fig. S1).

Out-of-sample predictive performance was assessed by approximate leave-one-out cross-validation using Pareto-smoothed importance sampling [31]. All Pareto-*k* diagnostics were below 0.7, indicating that the importance sampling approximation was reliable for every observation. Because the response is binary, we additionally report leave-one-out discrimination (area under the receiver operating characteristic curve), the Brier score, and a calibration plot of observed infection proportion against leave-one-out predicted probability in deciles of predicted risk (Additional file 1: Fig. S2). Predictive performance of each covariate model is reported relative to the null model, so that the incremental contribution of the covariates is explicit.

#### Spatial dependence

Spatial non-independence among individuals was accounted for by the site-level varying intercept, which allows infection probability to differ among sites without assuming spatial structure. Because that term treats sites as exchangeable and does not model distance-dependent similarity, we additionally tested for residual spatial autocorrelation. Site-level mean Pearson residuals were extracted from the fitted model and Moran’s *I* computed on an inverse-distance weights matrix. Residual autocorrelation was not detectable (Moran’s *I* = −0.049, expected −0.012, *P* = 0.25), indicating that variation among sites was not distance-structured, and no explicit spatial term was therefore included.

#### Reporting

Posterior distributions are summarized by the median and the 89% credible interval. In addition to coefficients on the log-odds scale, we report average marginal effects on the probability scale, computed by standardizing predictions over the observed sample with group-level effects marginalized out. We do not dichotomize results by whether an interval excludes zero; effects are described in terms of the direction and magnitude the posterior supports. The data and analysis code used in this study are publicly available in the corresponding GitHub repository at https://github.com/bhopalstiffs/CBRBatMalaria.

## Results

### Prevalence of haemosporidian parasite infections in Cambodian bats

A total of 1180 bats belonging to 34 species of seven bat families were screened for infections with haemosporidian parasites. The infections were molecularly verified in 152 individuals of eleven bat species representing four different bat families (Emballonuridae, Hipposideridae, Pteropodidae, and Vespertilionidae) corresponding to an overall prevalence of 12.9% (152/1180) (Table 1).

*Polychromophilus* infections were restricted to a small number of bat species, occurring in the vespertilionid species *Scotophilus kuhlii, S. heathii* and *Myotis rosseti,* and *Hipposideros diadema* (Hipposideridae). Prevalence differed markedly between the two *Scotophilus* species, with 54% (35/65) in *S. kuhlii* and 4.0% (1/25) in *S. heathii*, whereas infections in *M. rosseti* and *H. diadema* were each detected in one of two individuals (Table 1).

*Hepatocystis* infections were detected across multiple bat families (Emballonuridae, Hipposideridae, and Pteropodidae). Notably, infections in *Taphozous melanopogon* (18%, 15/83) represent the first record of *Hepatocystis* in Emballonuridae, substantially expanding the known host range of this parasite genus. Within Pteropodidae, infections were common in *Cynopterus brachyotis* (42%, 43/102) and *C. sphinx* (11%, 26/241) and were also detected in the newly recorded host species *Megaerops niphanae* and *Eonycteris spelaea*. In Hipposideridae, infections occurred in *H.* cf. *grandis* (23%, 26/114) and were additionally detected in *H. galeritus* and *H. diadema*.

**Fig. 2:**
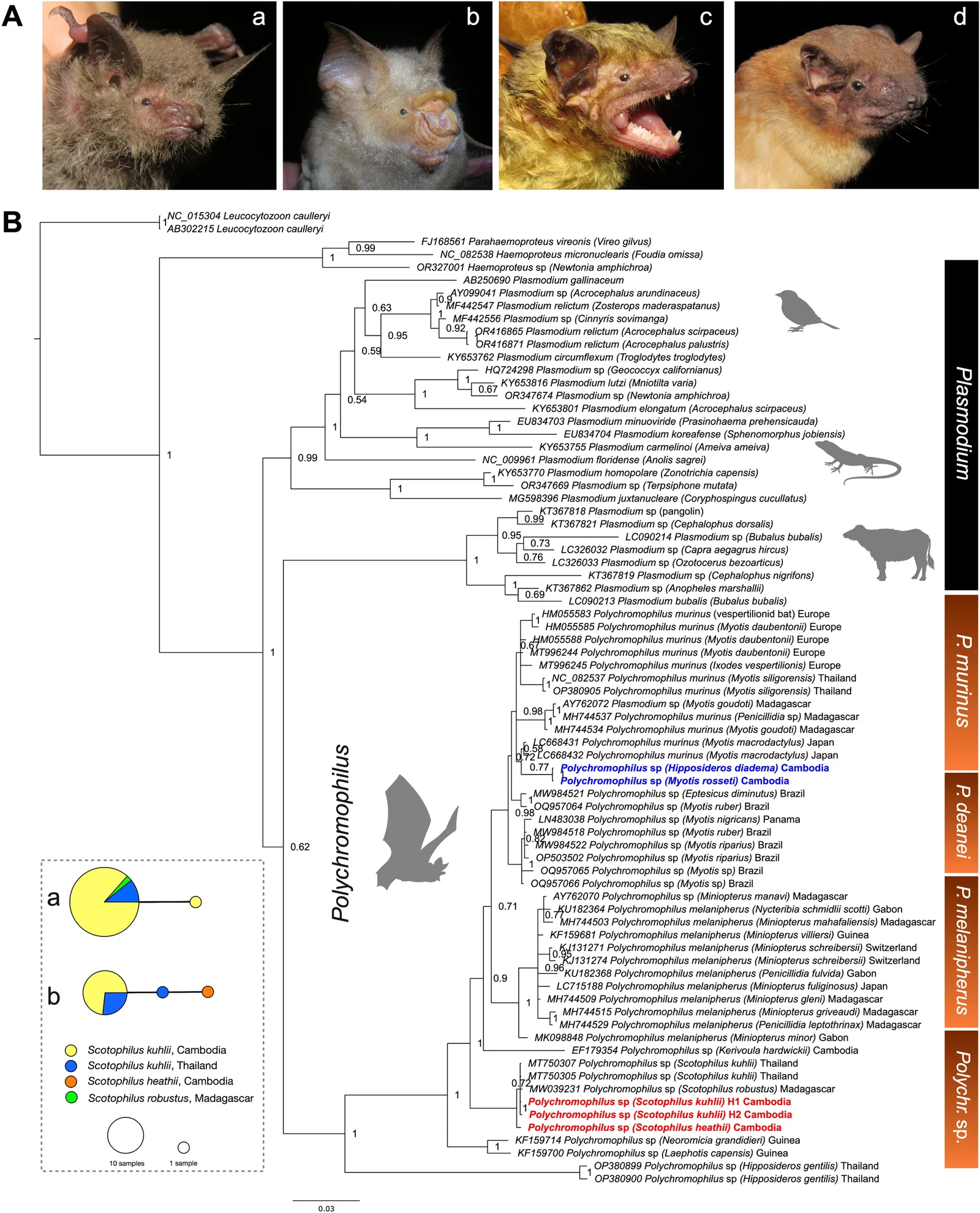
Characterization of *Polychromophilus* parasites in Cambodian bats. **A** Photographs (credit: Neil Furey) show the bat host species, (a) *Myotis rosseti, (b) Hipposideros diadema, (c) Scotophilus heathii, (d) Scotophilus kuhlii.* **B** Phylogeny of *Polychromophilus* parasites. Bayesian inference analysis based on a concatenated alignment (2541 nt) of mitochondrial sequences of *cytb* (1110 nt) and *cox1* (1,431 nt). Inset: Median-joining haplotype networks of all *Polychromophilus* sequences of *Scotophilus* host species, (**a**) *cytb* gene (front part: 474 nt, *n* = 38), (**b**) *cox1* gene (407 nt, *n* = 17). The line between haplotypes represents one base change, node size is proportional to the number of individuals assigned to each haplotype

**Fig. 3:**
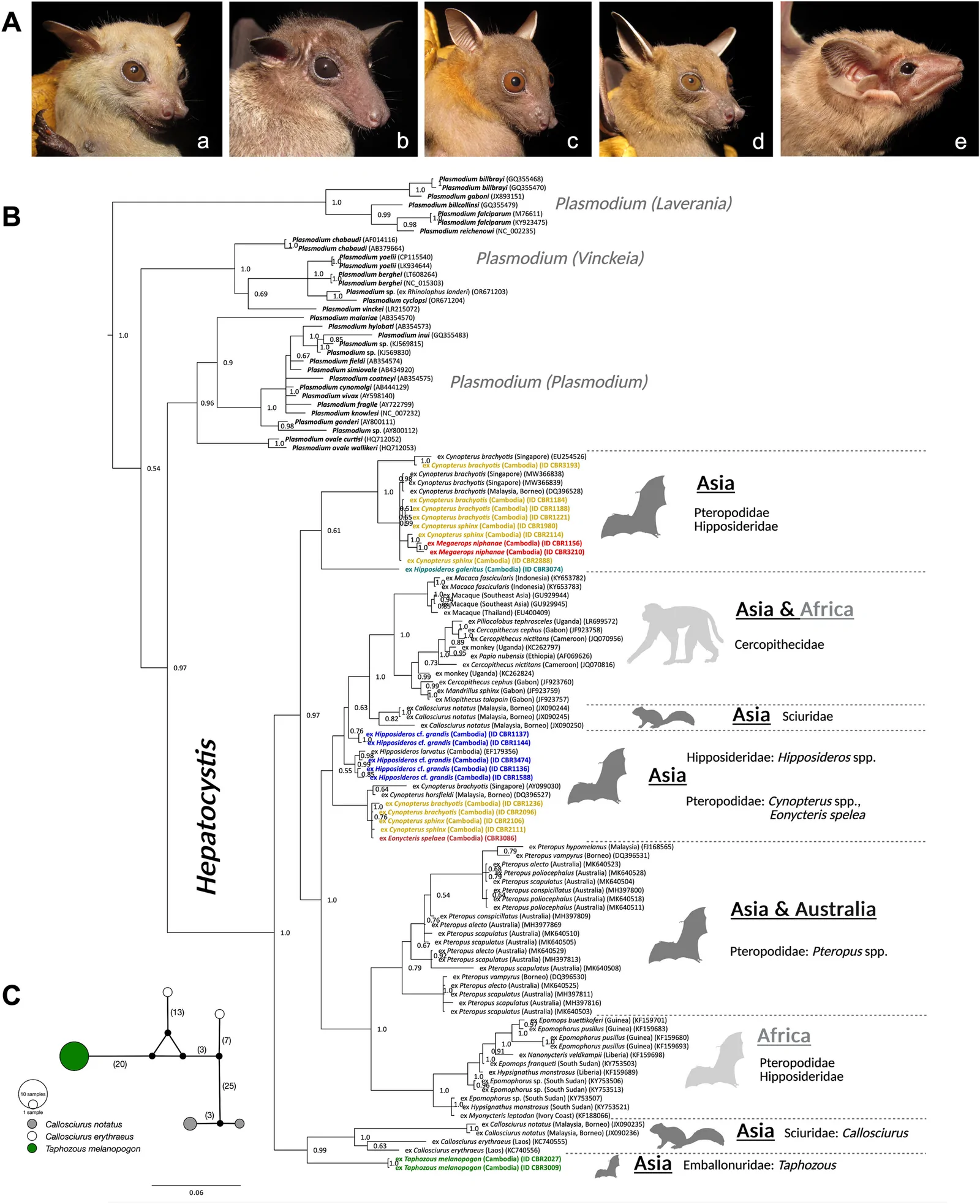
Phylogeny of *Hepatocystis* parasites. **A** Photographs (credit: Neil Furey) show the bat host species, (a) *Megaerops niphanae,* (b) *Eonycteris spelaea,* (c) *Cynopterus brachyotis,* (d) *Cynopterus sphinx,* (e) *Taphozous melanopogon.* **B** Bayesian inference analysis based on mitochondrial sequence alignment of *cytb* (927 nt). Sequences of the study are highlighted in bold and coloured according to bat host genus. Full tree is provided as Additional file 1: Fig. S4. **C** Median-joining haplotype networks of *Hepatocystis* parasites of *Taphozous* bat and *Callosciurus* species. Median-joining haplotype network of *cytb* (534 nt) sequences of *Hepatocystis* parasites isolated from the bat species *Taphozous melanopogon* (*n* = 10) and from the rodent (squirrel) species *Callosciurus erythraeus* (*n* = 2) and *C. notatus* (*n* = 2). The line between haplotypes or nodes represents one base change unless otherwise labelled in parentheses. Node size is proportional to the number of individuals assigned to each haplotype

**Fig. 4:**
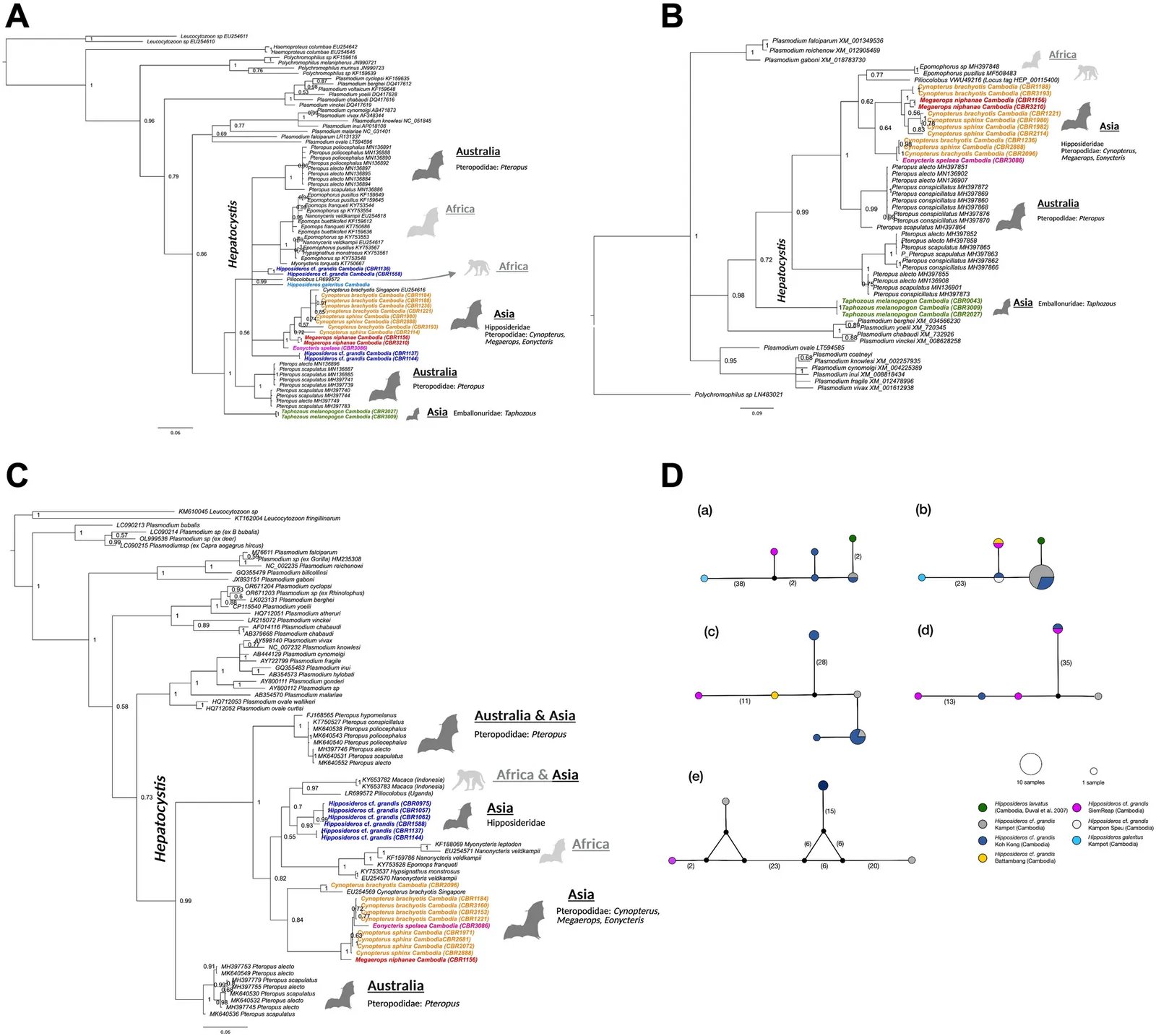
Evolutionary relationships of *Hepatocystis* parasites. Sequences of the study are highlighted in bold and coloured according to bat host genus. Bayesian inference analyses based on: **A** sequence alignment of the apicoplast *clpC* locus (504 nt), **B** sequence alignment of the nuclear *pat* locus (831 nt), **C** sequence alignment of mitochondrial *cox1* (915 nt). No reliable *cox1* sequences could be amplified for the *Taphozous*-*Hepatocystis* samples with the used PCR protocols. **D** Haplotype median-joining networks of *Hepatocystis* parasites of *Hipposideros* spp. bats: (a) *cytb* (681 nt, *n* = 7), (b) *cytb* (487 nt, *n* = 19), (c) *cox1* (640 nt, *n* = 11), (d) *clpC* (484 nt, *n* = 6), (e) *ef2* (529 nt, *n* = 5)

**Fig. 5:**
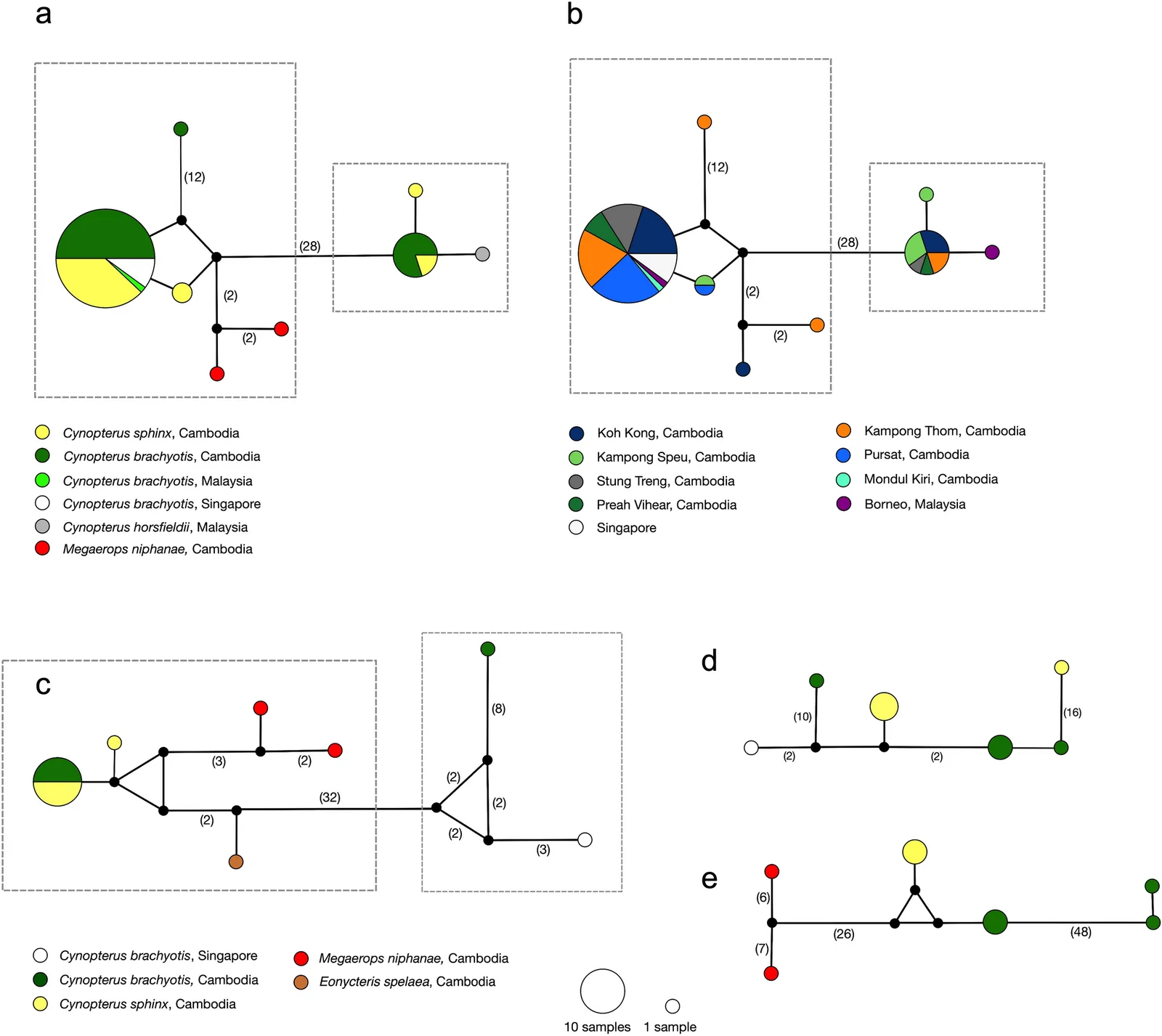
Median-joining haplotype networks of *Hepatocystis* parasites of *Cynopterus, Megaerops, Eonycteris,* and *Taphozous* bat species. **a** *cytb* (434 bp, *n* = 67) trait host species. **b** *cytb* (434 bp, *n* = 67) trait locality. **c** *cox1* (450 bp, *n* = 18) **d** *clpC* (496 bp, *n* = 11) **e** *ef2* (521 bp, *n* = 10). The line between haplotypes or nodes represents one base change unless otherwise labelled in parentheses. Node size is proportional to the number of individuals assigned to each haplotype

**Fig. 6:**
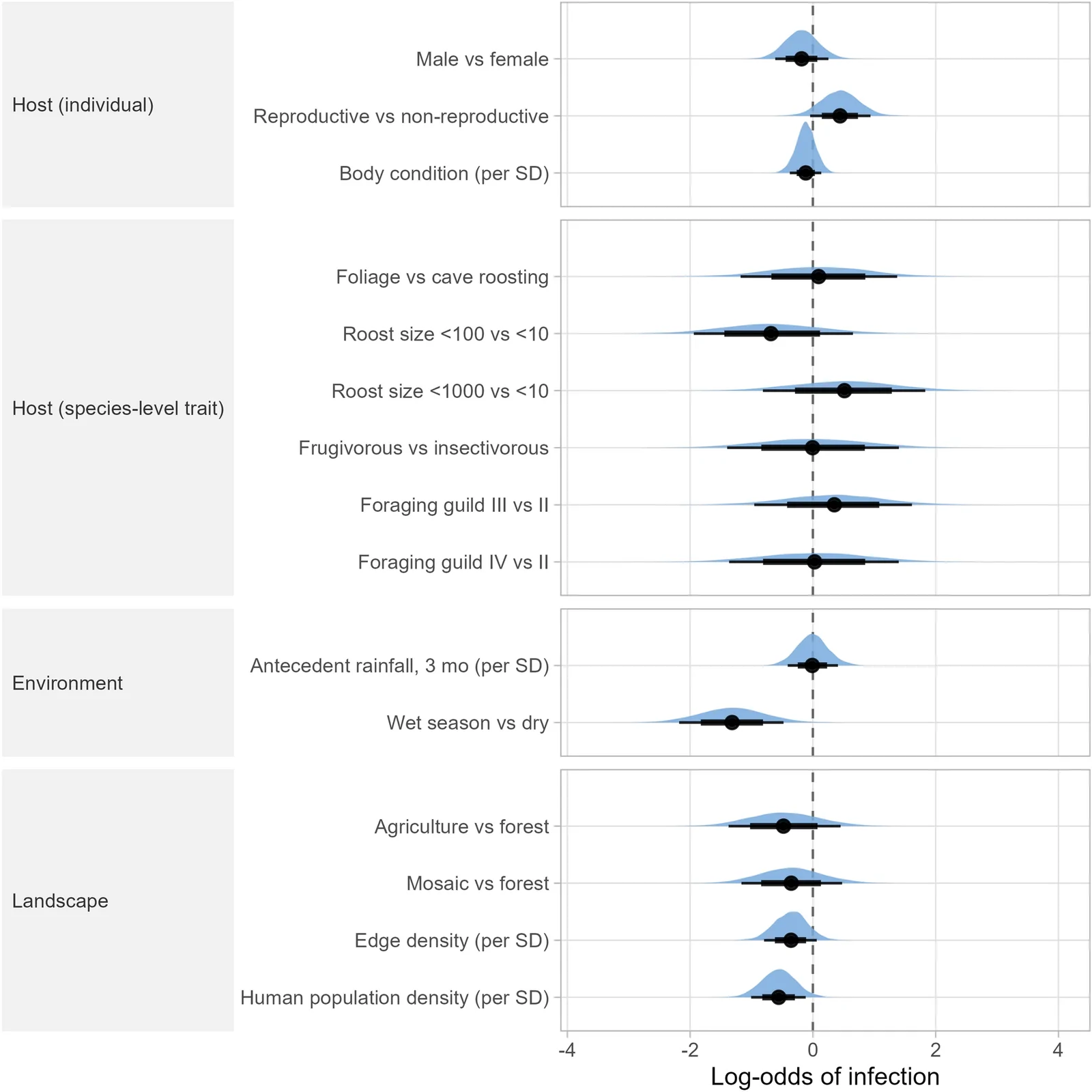
Population-level effects on haemosporidian infection probability in Cambodian bats. Posterior distributions of coefficients from the integrated Bernoulli generalized linear mixed model fitted to 589 individuals of 10 host species at 84 sites, with crossed varying intercepts for host species and sampling site. Point estimates are posterior medians; thick and thin bars show 66% and 89% credible intervals, respectively. Coefficients are on the log-odds scale; continuous covariates were standardized, so their coefficients refer to a change of one standard deviation. Panels group covariates by the level at which they vary. Coefficients for the four species-level traits are estimated from contrasts among the 10 host species sampled rather than among individuals, which accounts for their markedly wider intervals. Reference levels are given in Table 2. Blue densities show the posterior distributions

No haemosporidian infections were detected in bats from the families Molossidae, Megadermatidae, and Rhinolophidae. Rhinolophidae (n = 84 individuals) and Megadermatidae (16 individuals) were screened from 29 and 12 sites respectively, distributed across most of the latitudinal range of the survey, so the absence of infection in these families is unlikely to reflect restricted sampling. Molossidae were represented by 288 *Mops plicatus* screened at only eight sites in three provinces, reflecting the colonial roosting habits of the species, the absence of infection in this family therefore rests on a spatially concentrated sample. These families were therefore excluded from the subsequent ecological analyses, which included only bats belonging to host genera in which infections were confirmed (*n* = 663 individuals from 84 sites across 21 provinces, Fig. 1). Infections of *Polychromophilus* were restricted to fewer host taxa than *Hepatocystis,* which occurred across multiple bat families Fig. 2, 3, 4, 5 and 6.

### Ecological correlates of haemosporidian infection

We modelled infection status as a function of host, environmental, and landscape covariates in a single Bernoulli generalized linear mixed model with crossed varying intercepts for host species and sampling site. The analyzed sample comprised 131 infections among 589 individuals (22.2%) of 10 host species at 84 sites; 439 individuals were from probabilistically selected sites and 150 from opportunistically selected sites. Sampling effort per site ranged from 1 to 65 individuals (median 4).

**Fig. 7:**
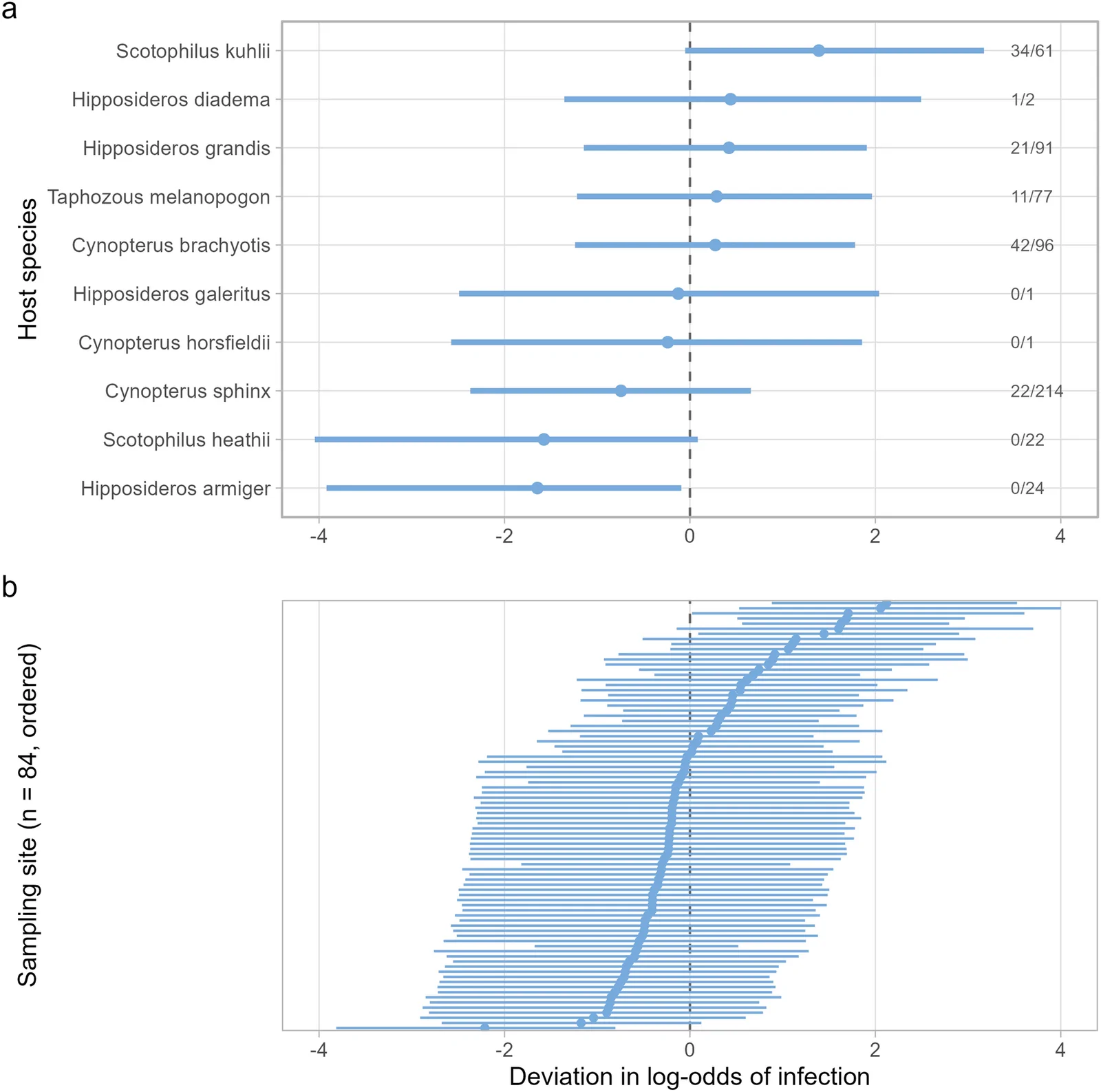
Group-level variation in haemosporidian infection probability among host species and sampling sites. Deviations from the population mean on the log-odds scale, from the integrated model described in Fig. 6; points are posterior medians and bars 89% credible intervals. **a** Host species, ordered by posterior median, with the number of infected individuals over the number screened shown at right. **b** The 84 sampling sites, ordered by posterior median and shown without labels; individual sites are not interpreted. Both panels share an x-axis scale. Host species and sampling site accounted for 26.3% (89% CrI 8.5–50.4%) and 25.3% (89% CrI 13.1–41.2%) of the latent-scale variance, respectively. Species and sites represented by few individuals are shrunk toward the population mean under partial pooling, so the ordering in both panels reflects sampling effort as well as observed prevalence

Infection probability varied more among host species and among sites than among individuals within them. On the latent scale, host species accounted for 26.3% of the total variance (89% CrI 8.5–50.4%) and that for sampling site 25.3% (89% CrI 13.1–41.2%). *Scotophilus kuhlii*, in which 34 of 61 individuals were infected, showed the highest species-level deviation, and *Hipposideros armiger*, in which no infections were detected among 24 individuals, the lowest, although no individual species intercept was clearly separated from the population mean (Fig. 7a). Variation among sites was not distance-structured (Moran’s *I* = −0.049, expected −0.012, *P* = 0.25), and no explicit spatial term was therefore required (Fig. 7b). Adding the measured covariates did not materially improve predictive performance beyond the null model containing the intercept and the host-species and sampling-site varying intercepts. Leave-one-out cross-validation gave no support for any covariate model over one containing only the intercept and the two varying intercepts, and discrimination was effectively identical across models (area under the receiver operating characteristic curve 0.829 for the intercept-only model and 0.832 for the full model; Additional file 1: Table S8, Fig. S2). Host species and capture site were therefore as informative as any combination of the covariates we measured.

Infection probability was substantially lower in the wet season than in the dry season (− 1.32, 89% CrI −2.18 to −0.48), the largest effect in the model. Cumulative rainfall over the three months preceding capture was not itself associated with infection (− 0.01, 89% CrI −0.41 to 0.41), and the two covariates were only weakly correlated (*r* = 0.36), indicating that the seasonal signal is not reducible to precipitation volume.

Infection probability declined with increasing human population density within a 2-km radius of the capture site (− 0.56, 89% CrI −1.00 to −0.12) and tended to decline with increasing edge density, although the interval included zero (− 0.36, 89% CrI −0.79 to 0.06). Land cover class at the site was not associated with infection probability (agriculture relative to forest −0.47, 89% CrI −1.37 to 0.45; mosaic relative to forest −0.35, 89% CrI −1.16 to 0.48).

Among individual-level host covariates, reproductive individuals were more likely to be infected than non-reproductive individuals, although the interval overlapped zero (0.44, 89% CrI −0.04 to 0.94). Neither host sex (− 0.18, 89% CrI −0.61 to 0.25) nor body condition (− 0.12 per SD, 89% CrI −0.38 to 0.14) was associated with infection probability.

Host traits recorded at the level of the species; roost type, typical roost size, feeding guild, and foraging guild, were not associated with infection probability and were estimated with very low precision (posterior SD 0.80–0.88 on the log-odds scale; Table 2). Because each of these traits is invariant within a host species, their effects are estimated from contrasts among the 10 species sampled rather than among the 589 individuals screened, and they compete with the species varying intercept for the same between-species variation. Resolving trait effects on haemosporidian infection would require a substantially larger number of host species rather than a larger number of individuals.

**Table 2:** Population-level effects on haemosporidian infection probability

Block	Predictor	Log-odds (89% CrI)	SD	Marginal effect, pp (89% CrI)
Host (individual)	Male vs female	−0.18 (− 0.61, 0.25)	0.27	– 2.1 (− 7.7, 3.1)
Host (individual)	Reproductive vs non-reproductive	0.44 (− 0.04, 0.94)	0.31	4.9 (− 0.5, 11.8)
Host (individual)	Body condition (per SD)	– 0.12 (− 0.38, 0.14)	0.16	– 1.3 (− 4.4, 1.7)
Host (species-level trait)	Foliage vs cave roosting	0.09 (− 1.18, 1.37)	0.81	1.0 (− 15.4, 15.7)
Host (species-level trait)	Roost size <100 vs <10	– 0.66 (− 1.94, 0.65)	0.81	– 7.4 (− 24.4, 7.4)
Host (species-level trait)	Roost size <1000 vs <10	0.51 (− 0.81, 1.83)	0.83	7.1 (− 11.4, 27.2)
Host (species-level trait)	Frugivorous vs insectivorous	0.00 (− 1.40, 1.40)	0.88	– 0.1 (− 16.4, 17.9)
Host (species-level trait)	Foraging guild III vs II	0.33 (− 0.95, 1.61)	0.80	3.9 (− 11.2, 21.5)
Host (species-level trait)	Foraging guild IV vs II	0.02 (− 1.36, 1.40)	0.87	0.3 (− 16.1, 17.0)
Environment	Antecedent rainfall, 3 mo (per SD)	– 0.01 (− 0.41, 0.41)	0.25	– 0.1 (− 4.6, 5.4)
Environment	Wet season vs dry	– 1.32 (− 2.18, − 0.48)	0.53	– 14.7 (− 27.7, − 4.8)
Landscape	Agriculture vs forest	– 0.47 (− 1.37, 0.45)	0.57	– 5.6 (− 18.1, 5.6)
Landscape	Mosaic vs forest	– 0.35 (− 1.16, 0.48)	0.51	– 4.3 (− 15.4, 5.9)
Landscape	Edge density (per SD)	– 0.36 (− 0.79, 0.06)	0.27	– 3.8 (− 8.8, 0.8)
Landscape	Human population density (per SD)	– 0.56 (− 1.00, − 0.12)	0.28	– 5.7 (− 10.9, − 1.2)

Reference levels: female (sex), non-reproductive (reproductive status), cave (roost type), <10 (typical roost size), insectivore (feeding guild), guild II (foraging guild), dry (season), forest (land cover class). Posterior median, standard deviation, and 89% credible interval on the log-odds scale, with the average marginal effect on the probability scale (percentage points). Estimates are from the integrated model fitted to 589 individuals of 10 host species at 84 sites, with crossed varying intercepts for host species and sampling site. Continuous covariates are standardized, so their coefficients refer to a change of one standard deviation

#### Sensitivity analyses

Estimates were robust to the analytical choices we examined (Additional file 1: Tables S4 and S6–S8, Figs S1–S2). Adding a term for sampling design attenuated the population density coefficient slightly but did not change its direction or the strength of support for it (− 0.48, 89% CrI − 0.94 to − 0.02). The design term itself was poorly resolved (− 0.78, 89% CrI − 1.93 to 0.38). Extracting landscape covariates within a 5-km rather than a 2-km buffer gave comparable estimates: population density remained negative (− 0.62, 89% CrI − 1.18 to − 0.08) and edge density remained negative but less precisely estimated (− 0.28, 89% CrI − 0.76 to 0.19). Estimates under an alternative specification with priors centred away from zero for body condition, edge density, and population density were similar for most covariates but more strongly negative for the latter two, as expected given the direction of those priors. All models converged (R̂ ≤ 1.00, minimum bulk effective sample size 2097, minimum tail effective sample size 3502) with no divergent transitions.

### Phylogenetic relationships of *Polychromophilus* parasites

Phylogenetic analyses confirmed *Polychromophilus* as sister taxon of ungulate-infecting *Plasmodium* species [32] (Fig. 2). *Polychromophilus* sequences from hipposiderid bats in Thailand [33] formed a basal clade distinct from the remaining *Polychromophilus* lineages, and parasites from vespertilionid bats in Guinea also grouped as a separate clade [13]. Within the genus, *Polychromophilus melanipherus* formed a monophyletic clade, whereas *Polychromophilus murinus* sequences from Old World bats clustered with Neotropical vespertilionid bat parasites, likely representing* Polychromophilus deanei.*

*Polychromophilus* parasites detected in *Hipposideros diadema* and *Myotis rosseti* shared identical haplotypes (*cytb, cox1*) and grouped closely with sequences of *P. murinus.* In contrast, *Polychromophilus* sequences from *S. kuhlii and S. heathii* bats formed a well-supported clade (pp = 1.0) comprising only *Polychromophilus* parasites from *Scotophilus* spp. hosts (Fig. 2). These sequences did not group with the only previously reported *Polychromophilus* from a bat in Cambodia that had been isolated from *Kerivoula hardwickii* [34]*.*

*Polychromophilus* associated with *Scotophilus* spp. form a monophyletic lineage including sequences from *S. kuhlii, S. heathii* and *Scotophilus robustus*, with origins in Cambodia, Thailand and Madagascar. The haplotype network analyses of the front part of *cytb* (474 nt, n = 38) that comprises all *Polychromophilus* sequences from *S. kuhlii* bats of Cambodia, Thailand and from the host species *S. robustus* from Madagascar revealed one shared haplotype (Fig. 2Ba). One additional haplotype was recovered for a sequence isolated from *S. kuhlii* from Cambodia (sample was collected in a managed pine forest (plantation) in Kampong Chhnang) that features one base change to the main haplotype (Fig. 2Ba). The parasite sequence from *S. heathii* could only be generated for the back part of *cytb* which shares no overlap with the parasite *cytb* sequences of Thailand and Madagascar, but with those of *S. kuhlii* of Cambodia which resulted in two distinct haplotypes that only differ by one base (392 nt, n = 12) (Additional file 1: Fig. S3). Overall, haplotype network analyses across *cytb, cox1, clpC*, and *ef2* revealed extremely low genetic diversity among *Scotophilus*-associated parasites, with identical or near-identical haplotypes across Cambodia, Thailand, and Madagascar (Fig. 2B a, b; Additional file 1: Fig. S3).

### Phylogenetic relationships of *Hepatocystis* parasites

Bayesian analysis of mitochondrial *cytb* sequences confirmed *Hepatocystis* as a monophyletic group closely related to mammalian *Plasmodium* (*Plasmodium*), consistent with previous studies [13] (Fig. 3). Other analyses based on nuclear datasets suggest a closer relationship to the *Plasmodium* (*Vinckeia*) clade [5, 35]. Within this group, parasite sequences from Cambodian bats are recovered within several different clades*,* indicating substantial lineage diversity.

*Hepatocystis* parasites from the emballonurid bat *Taphozous melanopogon* (Fig. 3A) were highly conserved, with one haplotype across different genetic markers (*cytb*, 534 nt, n = 10; c*lpC*, 518 nt, n = 14). Notably, *cytb* parasite sequences from *T. melanopogon* formed a well-supported clade with *Hepatocystis* lineages infecting Southeast Asian squirrels (*Callosciurus* spp.) (pp = 0.99) (Fig. 3B) despite substantial mitochondrial divergence between host associated groups (Fig. 3C). Three additional parasite sequences from *Callosciurus notatus* (Malaysia) grouped outside this clade and instead clustered with primate-associated *Hepatocystis* lineages (Fig. 3B).

Phylogenetic analyses based on additional loci (*clpC*, *pat*) recovered the *Taphozous*-associated lineage as basal to other *Hepatocystis* taxa included in these datasets, although interpretation is limited by the absence of comparable rodent-derived sequences (Fig. 4A, B).

More broadly, the *cytb* phylogeny revealed multiple well-supported subgroups within *Hepatocystis.* One group comprised parasites from primate hosts (Cercopithecidae), structured into Asian and African subclades, with two *Callosciurus-*derived sequences basal to this group. Two additional monophyletic clades formed a strongly supported sister group (pp = 1.0), comprising *Hepatocystis* lineages from Asian and Australian *Pteropus* hosts and from African pteropodid bats, respectively.

*Hepatocystis* parasites infecting hipposiderid bats were phylogenetically diverse and did not form a single clade (Fig. 3B, Fig. 4A, C). Parasites from *H.* cf*. grandis* occurred in two distinct lineages, both basal to the *Callosciurus*/primate-associated group. One of these lineages included a previously published sequence from *Hipposideros larvatus* in Cambodia [34], which differed by two nucleotides in the *cytb* haplotype network (Fig. 4Da). Overall, haplotype network analyses across multiple loci (*cytb*, *cox1, clpC, ef2*; Fig. 4D) confirmed that *Hepatocystis* lineages from *Hipposideros* hosts are genetically diverse and do not cluster to geographic region. The parasite isolated from *H. galeritus* was particularly divergent, forming a distinct haplotype and showing weak phylogenetic affinity to lineages infecting Asian fruit bats (Pteropodidae) (pp = 0.61) (Fig. 3, Fig. 4D).

The largest dataset comprised *Hepatocystis* parasites from *Cynopterus brachyotis* and *C. sphinx*. These lineages were not recovered as a monophyletic group but instead formed two deeply divergent clades that were not structured by host species or geography (Fig. 3). The two clades differed by 30–41 nucleotide substitutions in *cytb* (434 nt, *n* = 67) and included parasites from additional bat species (*Megaerops niphanae* and *Eonycteris spelaea*) (Fig. 3, Fig. 4, Fig. 5). Although our host identifications were based on standard morphological criteria and verified by barcoding, some degree of misidentification among the closely related *Cynopterus* species (particularly juveniles) cannot be fully excluded; however, such uncertainty is unlikely to explain the deep mitochondrial divergence observed between the two *Hepatocystis* lineages we recovered.

Analyses of additional loci (*cox1, clpC, ef2, pat*) recovered *Hepatocystis* sequences from *Cynopterus*, *Eonycteris,* and *Megaerops* hosts as a single broader clade, with *Eonycteris* and *Megaerops* lineages basal to *Cynopterus* parasites in most analyses. The *pat* dataset further resolved two *Cynopterus*-associated sister clades, each including sequences from *Eonycteris* or *Megaerops*, respectively (Fig. 4, Fig. 5).

To further assess the placement of Southeast Asian bat-associated *Hepatocystis* lineages, we incorporated additional *cytb* sequences from Thai bats [36]. Due to the short length of these sequences (378 nt), the resulting phylogeny showed limited resolution and multiple polytomies, preventing robust inference of relationships among major clades. However, haplotype network analysis (375 nt, n = 111) recovered sequences from *Rhinolophus pearsonii* within clusters of *Hepatocystis* lineages infecting *Hipposideros* hosts (Additional file 1: Fig. S5).

## Discussion

Our study reveals substantial haemosporidian diversity in Cambodian bats, including multiple novel host associations and contrasting patterns of host specificity and ecological correlates between *Polychromophilus* and *Hepatocystis*. We confirmed a highly conserved, host-restricted lineage in *Polychromophilus* in *Scotophilus* bats, whereas *Hepatocystis* comprised diverse lineages across multiple bat taxa. Using a large multi-host dataset and multilocus phylogenetic analyses, we detected several previously undocumented host associations, including the first record of *Hepatocystis* in Emballonuridae, and recovered phylogenetic relationships consistent with historical host transitions among mammalian groups. Together, these findings expand current understanding of bat haemosporidian diversity, host associations, and evolutionary relationships, and further highlight bats as an important system for studying the diversification of mammalian malaria parasites.

Our results substantially expand sampling of a previously described *Scotophilus*-associated *Polychromophilus* lineage and support its remarkable genetic conservation across broad geographic regions [37, 38]. The extremely low genetic diversity observed across loci suggests either recent dispersal or long-term evolutionary conservatism. Together, these findings indicate strong host specificity at the host genus level, which is notable given the wide geographic distribution of this *Polychromophilus* lineage in *Scotophilus* bats, spanning regions such as Madagascar and Cambodia. In addition, this lineage forms a well-supported monophyletic group, does not cluster with currently recognized *Polychromophilus* species, and appears restricted to *Scotophilus* hosts. These characteristics may indicate a distinct evolutionary lineage with potential taxonomic significance, although morphological and additional molecular data will be required to clarify its status within *Polychromophilus*.

Species of *Polychromophilus* differ in host range and geographic distribution. *Polychromophilus melanipherus* is typically associated with bats of the family Miniopteridae and is considered panmictic, showing little to no geographic structuring across the ‘Old World’ [38, 39]. *Polychromophilus murinus* is most commonly reported from vespertilionid bats across Europe, Africa and Asia [4], whereas *Polychromophilus deanei* is largely restricted to vespertilionids in the Americas [40, 41]. Molecular studies indicate that *Polychromophilus* diversity and host associations are greater than currently recognized [13, 34, 37, 39]. The diversity and host associations of *Polychromophilus* parasites are of particular interest because this genus is exclusively associated with bats and represents the only haemosporidian lineage infecting bats across both temperate and tropical regions worldwide [4].

*Hepatocystis*, a parasite genus closely related to mammalian *Plasmodium*, provides a key evolutionary link for understanding malaria parasite diversification in mammals. The lineages identified in Cambodian bats provide new information on the unresolved phylogenetic relationships within this group, which infects diverse mammalian hosts including bats, primates, ungulates, and rodents [1]. Several morphospecies of *Hepatocystis* have been described from Asian bats, but molecular data remain sparse and are rarely linked to morphology (Additional file 1: Table S10). In our study, parasites detected in *Hipposideros galeritus*, *H.* cf*. grandis*, *Cynopterus brachyotis,* and *C. sphinx* could not be confidently assigned to described morphospecies due to the absence of blood-smear data. Nevertheless, the molecular data reveal substantial lineage diversity. In particular, parasites from *H.* cf*. grandis* occur in two distinct lineages, and *Hepatocystis* sequences from *Cynopterus* hosts fall into two deeply divergent clades that are not structured by host species. These findings suggest that host specificity in Asian bat *Hepatocystis* may be more limited than implied by the classical morphology-based taxonomy, or that currently recognized morphospecies may include cryptic diversity, as previously suggested for *Hepatocystis epomophori* in African bats [42]. Morphological data are therefore needed to complement the molecular analyses and refine species delineation.

A particularly notable result is the detection of *Hepatocystis* in *Taphozous melanopogon*, representing the first record of this parasite genus in Emballonuridae*.* Although this bat family is widely distributed, haemosporidian infections have rarely been documented in these hosts [36, 43]. In the mitochondrial phylogeny, the *Taphozous*-associated parasites cluster with *Hepatocystis* lineages from Southeast Asian squirrels, whereas analyses of additional loci (that lack reference sequences of squirrel-associated *Hepatocystis*) recover the *Taphozous* lineage as basal to the remaining *Hepatocystis* taxa included in those datasets. Although these relationships should be interpreted cautiously given the limited availability of multilocus reference data, they suggest a potentially important role for Asian non-pteropodid bats and/or rodents in the evolutionary history of *Hepatocystis.* Our findings therefore suggest that the evolutionary history of *Hepatocystis* may be more complex than previously proposed and may involve multiple historical host transitions among mammalian groups.

The close relationship between *Taphozous-*derived and squirrel-derived *Hepatocystis* lineages is consistent with historical host transitions between mammalian groups. Similar patterns have been observed in *Plasmodium (Vinckeia)* parasites, where phylogenetic proximity between rodent- and bat-associated lineages has been linked to ecological overlap and shared exposure to dipteran vectors [13, 44]. Although vectors of bat-associated *Hepatocystis* remain unknown, available evidence implicates *Culicoides* midges (Ceratopogonidae) [45–47], whose broad host-feeding behaviour [48] could facilitate cross-host transition opportunities. Further work integrating vector ecology and expanded sampling will be required to evaluate these hypotheses.

More broadly, our phylogenetic results suggest that Asian bats and rodents harbour substantial *Hepatocystis* diversity distributed across distantly related clades, whereas African bat and primate-associated lineages each form monophyletic clades. Although uneven taxon sampling may contribute to this pattern, the available data suggest that substantial diversification within *Hepatocystis* may have occurred among Asian mammalian hosts. This hypothesis will require testing with broader taxon sampling, particularly from Asian rodents and additional bat families, and ideally with integrated morphological and multilocus molecular data. Future work should also target *Hepatocystis* parasites from poorly sampled mammalian groups, including chevrotains and hippopotamuses, to better resolve the deeper evolutionary history of the genus. Beyond parasite diversity and evolutionary relationships, our analyses also revealed ecological correlates of infection in bats.

Our ecological analyses indicate that haemosporidian occurrence in Cambodian bats varies more among host species and among roosting sites than with any of the host traits or landscape variables we measured. Host species and sampling site together accounted for approximately half of the variation in infection probability and knowing an individual’s species and capture location predicted infection as well as any combination of measured covariates. Infection risk in this system therefore appears to be structured principally by host identity and by local conditions at the roost, rather than by the broader-scale landscape attributes.

The strongest effect we detected was seasonal: infection probability was substantially lower in the wet season than in the dry season. This direction is notable, since the wet season (May–October) is when *Culicoides* larval habitat should be most available, and a simple vector-abundance explanation would predict the opposite. Cumulative rainfall over the 3 months preceding capture showed no comparable association, indicating that the seasonal pattern is not a function of precipitation volume. Season may instead index vector phenology, temperature, or the timing of host reproduction, each of which could plausibly influence transmission. For *Polychromophilus*, nycteribiid bat fly abundance can vary seasonally [49], and for *Hepatocystis*, *Culicoides* emergence depends on the availability and persistence of larval habitat rather than total rainfall [50]. Seasonal shifts in host demography may also contribute: if parturition is timed to the onset of the wet season, the age and reproductive composition of captured bats will differ between seasons. Distinguishing among these possibilities will require vector sampling and bloodmeal analysis alongside host screening, which our study design did not include. Infection probability declined with increasing human population density. This is consistent with reduced availability of vector habitat in more densely populated landscapes: *Culicoides* midges, the presumed vectors of bat-associated *Hepatocystis,* depend on aquatic and semi-aquatic larval sites that are less available in built-up areas, and their limited dispersal range means transmission should be strongly influenced by conditions close to the roost [50–52]. Infection probability also tended to decline with increasing habitat fragmentation, although this association was weaker and the credible interval included zero; disrupted host-vector contact or reduced vector persistence in fragmented landscapes offers a plausible explanation [53], but our data do not establish the effect. Land cover class at the survey site showed no association with infection.

Among the individual-level host attributes, host sex had no detectable influence on infection probability, consistent with previous work [54]. Reproductive individuals were somewhat more likely to be infected, although the credible interval included zero; physiological or behavioural changes associated with reproduction have been proposed to influence susceptibility in other bat systems [55], and our data are consistent with, but do not confirm, such an effect. Body condition showed no association with infection probability. The consequences of haemosporidian infection for bats remain poorly understood, and previous studies have reported mixed results, with some evidence for elevated parasitaemia in younger individuals [56] but no consistent relationship with condition [57]. Given the distinctive immune biology of bats [58], the fitness consequences of infection remain difficult to assess and warrant further investigation.

The most striking host-level pattern was the contrast between the two *Scotophilus* species: *Polychromophilus* was detected in 34 of 61 *S. kuhlii* but in none of the 22 *S. heathii* screened. This difference is descriptive rather than a fitted effect, but it invites a roost-ecology explanation. In Cambodia, *S. kuhlii* commonly inhabits man-made structures used for guano collection, which may provide stable environments supporting persistent populations of nycteribiid bat flies, obligate ectoparasites with limited dispersal that depend on direct contact among hosts, while the continuous presence of infected bats may help sustain parasite transmission [59–63]. *S. kuhlii* has been reported to co-roost with other bat species in Southeast Asia, whereas there is no comparable evidence for *S. heathii,* nor that the two species co-roost in Cambodia. Opportunities for interspecific transfer of ectoparasites, and hence for *Polychromophilus* transmission, may therefore be limited for *S. heathii* under local conditions. Testing this would require direct observation of roost composition and ectoparasite loads.

Our capacity to resolve effects of host traits was limited by the number of host species rather than by the number of individuals screened. Roost type, typical roost size, feeding guild, and foraging guild are invariant within a species, so their effects are estimated from contrasts among the 10 species sampled, and all were estimated too imprecisely to support interpretation. Resolving how these traits shape haemosporidian infection will require datasets spanning substantially more host species. The same constraint applies to comparisons between parasite genera: because *Polychromophilus* and *Hepatocystis* occupied disjoint sets of host genera in our data, any apparent ecological difference between the parasites is equally a difference between their hosts, and the two cannot be separated. The studies designed to sample both parasite genera across shared host taxa would be needed to distinguish them. Together, these results indicate that fine-scale roost conditions and vector ecology, rather than landscape composition, are the more promising targets for understanding haemosporidian transmission in bats.

## Conclusions

Our results provide new insight into the diversity and evolutionary relationships of haemosporidian parasites in bats, including multiple novel host associations and the first record of *Hepatocystis* in Emballonuridae. *Scotophilus-*associated *Polychromophilus* forms a conserved, host-restricted lineage spanning Cambodia, Thailand, and Madagascar, whereas *Hepatocystis* comprises diverse lineages across multiple bat families and shows phylogenetic affinities with parasites of Asian rodents and primates. Our ecological analyses indicate that infection is structured principally by host species and by conditions at individual roosting sites, together with a pronounced seasonal difference in infection probability, rather than by the landscape attributes we measured. Resolving how host traits and vector ecology shape these patterns will require sampling across a wider range of host species, paired collection of vectors, and integration of morphological with multilocus molecular data. Such work would substantially advance understanding of how mammalian malaria parasites diversify and persist across host groups.

## Supplementary Information

Additional file 1: Fig. S1. Posterior predictive checks for the integrated model. Fig. S2. Leave-one-out calibration of predicted infection probability. Fig. S3. Median-joining haplotype networks of all *Polychromophilus* sequences of *Scotophilus* host species. Fig. S4. Phylogeny of *Hepatocystis* parasites. Fig. S5. *Cytb-*Haplotype median-joining network of *Hepatocystis* spp. Table S1. List of Oligonucleotides used in this study. Table S2. Overview of PCR temperature protocols of the study. Table S3. Information for Bayesian inference analyses. Table S4. Scale sensitivity. Table S5. Sampling design balance. Table S6. Prior sensitivity. Table S7. Sampler diagnostics. Table S8. Model comparison by approximate leave-one-out cross-validation. Table S9. Integrated model including a term for sampling design. Table S10. Overview of *Hepatocystis parasite* records from Asian bats.

Additional file 2: Dataset S1. Nucleotide sequence alignments

## Data Availability

All sequence data have been deposited in GenBank under the following accession numbers: PX831502–PX831548 (Hepatocystis, cytb), PX831549–PX831553 (Polychromophilus, cytb), PX831554–PX831571 (ef2), PX831627–PX831631 (Polychromophilus, cox1), PX831632–PX831637 (Polychromophilus, clpC), PX831638–PX831653 (Hepatocystis, clpC), PX831654–PX831678 (Hepatocystis, cox1), PX831679–PX831690 (Hepatocystis, pat). Supplementary information includes analyses tables and figures, phylogenetic trees, haplotype network figures and sequence alignments. The data and analysis code used in this study are publicly available in the corresponding GitHub repository at https://github.com/bhopalstiffs/CBRBatMalaria.
