# Supplementary material for: High diversity and novel host associations of bat haemosporidian parasites in Cambodia: insights from a nationwide bat survey and multilocus analyses
Source: Parasit Vectors. 2026 Oct 8;19:436. doi: 10.1186/s13071-026-07732-3 (PMC13651332; doi:10.1186/s13071-026-07732-3)
Supplement: Supplementary file 1 [file 13071_2026_7732_MOESM1_ESM.docx]

**Supplementary information**

**High diversity and novel host associations of bat haemosporidian parasites in Cambodia: insights from a nationwide bat survey and multilocus analyses**

Juliane Schaer, Oskar Werb, Lena Ch´ng, Dolyce Hong Wen Low, Sophie Alison Borthwick, Neil Furey, Liesbeth Frias, Awatsaya Pimsai, Raksmey Yim, Kimashalen Chor, Dany Cheang, Gavin JD Smith, Alan Hitch, Ian H Mendenhall

Figure S1. Posterior predictive checks for the integrated model

Figure S2. Leave-one-out calibration of predicted infection probability

Figure S3. Median-joining haplotype networks of all *Polychromophilus* sequences of *Scotophilus* host species

Figure S4. Phylogeny of *Hepatocystis* parasites (*cytb* full tree)

Figure S5. *Cytb-*Haplotype median-joining network of *Hepatocystis* spp.

Table S1. List of Oligonucleotides used in this study

Table S2. Overview of PCR temperature protocols of the study

Table S3. Information for Bayesian inference analyses

Table S4. Scale sensitivity

Table S5. Sampling design balance

Table S6. Prior sensitivity

Table S7. Sampler diagnostics

Table S8. Model comparison by approximate leave-one-out cross-validation

Table S9. Integrated model including a term for sampling design

Table S10. Overview of *Hepatocystis parasite* records from Asian bats

Figure S1. Posterior predictive checks for the integrated model. Observed counts of infected and uninfected individuals (dark) against 100 draws from the posterior predictive distribution (light), shown *a* overall, *b* by host species, and *c* by land cover class at the capture site. Grouped checks test whether the model reproduces the clustering of infection that the varying intercepts are intended to capture.

Figure S2. Leave-one-out calibration of predicted infection probability. Observed infection proportion against leave-one-out predicted probability, in deciles of predicted risk; bars are 95% binomial intervals, and the dashed line is the identity. Calibration assesses whether predicted probabilities are correct in absolute terms, which is distinct from the discrimination reported in Table S8.

Figure S3. Median-joining haplotype networks of all *Polychromophilus* sequences of *Scotophilus* host species. (a) *cytb* gene (front part: 474 nt, n = 38), (b) *cytb* gene (back part: 392 nt, n = 12), (c) *cox1* gene (407 nt, n = 17), (d) *clpC* gene (405 nt, n = 9). The line between haplotypes represents one base change, node size is proportional to the number of individuals assigned to each haplotype.

One additional haplotype was recovered for a sequence isolated from *S. kuhlii* from Cambodia (sample was collected in a managed pine forest (plantation) in Kampong Chhnang) that features one base change to the main haplotype. The parasite sequence from *S. heathii* could only be generated for the back part of *cytb* which shares no overlap with the parasite *cytb* sequences of Thailand and Madagascar, but with those of *S. kuhlii* of Cambodia which resulted in two distinct haplotypes that only differ by one base (392 nt, n = 12). The *cox1* haplotype network (407nt, n = 17) recovered three different haplotypes: one main haplotype with parasites sequences from *S. kuhlii* of Cambodia and Thailand that differs by one base from haplotype 2 (one parasite of *S. kuhlii* from Thailand) which again differs by one base to haplotype 3, the sequence of the *S. heathii* parasite. The c*lpC* haplotype network recovered the parasite sequence of *S. kuhlii* of Thailand as unique haplotype that differs by one and two bases from two haplotypes recovered from *S. kuhlii* of Cambodia, respectively (405 nt, n = 9). The *ef2* haplotype network (not shown) recovered the parasite sequence of *S. kuhlii* of Thailand as unique haplotype that differs by two bases from the haplotype recovered from *S. kuhlii* of Cambodia, respectively (348 nt, n = 6). One parasite sample from *S. kuhlii* (CBR0469) featured a mixed haplotype infection as several ambiguities were detected in the sequences (*cox1, clpC)*.

Figure S4. Phylogeny of *Hepatocystis* parasites (*cytb* full tree). Bayesian inference analysis based on mitochondrial sequence alignment of *cytb* (927 nt). Sequences of the study are highlighted in bold and colored according to bat host genus.

Figure S5. *Cytb-*Haplotype median-joining network of *Hepatocystis* spp. Haplotype network based on a short *cytb-*alignment (375 nt) to infer *Hepatocystis* relationships with published *cytb*-sequences (375 nt) from bats in Thailand (n = 111).

Table S1. List of Oligonucleotides used in this study

| **Gene** | **Primer name** | **Sequence (5´- 3´)** | **Reference** |
| --- | --- | --- | --- |
| *cytb* (mitochondrial) | Hep-F3 | CTTACCTTGGGGACAAATGAGTTATT | Schaer et al. 2013 |
|  | Hep-R3 | CTCTAGCACCAAATGTCATTTTAAATTG | Schaer et al. 2013 |
|  | DW2 | TAATGCCTAGACGTATTCCTGATTATCCAG | Perkins & Schall 2002 |
|  | DW4 | TGTTTGCTTGGGAGCTGTAATCATAATGTG | Perkins & Schall 2002 |
|  | 3932-F | GGGTTATGTATTACCTTGGGGTC | Perkins & Schall 2002 |
|  | 3932-R | GACCCCAAGGTAATACATAACCC | Perkins & Schall 2002 |
| *cox1*  (mitochondrial) | Cox1-F | CTATTTATGGTTTTCATTTTTATTTGGTA | Martinsen et al. 2008 |
|  | Cox1-R | AGGAATACGTCTAGGCATTACATTAAATCC | Martinsen et al. 2008 |
|  | Cox-in-F | ATGATATTTACARTTCAYGGWATTATTATG | Martinsen et al. 2008 |
|  | Cox-in-R | GTATTTTCTCGTAATGTTTTACCAAAGAA | Martinsen et al. 2008 |
| *clpC*  (apicoplast) | clpC-out-F | AAACTGAATTAGCAAAAATATTA | Martinsen et al. 2008 |
|  | clpC-out-R | CGWGCWCCATATAAAGGAT | Martinsen et al. 2008 |
|  | clpC-in-F | GATTTGATATGAGTGAATATATGG | Martinsen et al. 2008 |
|  | clpC-in-R | CCATATAAAGGATTATAWG | Martinsen et al. 2008 |
| *ef2* (nuclear) | EF2-F | GTTCGTGAGATCATGAACAAAAC | Schaer et al. 2013 |
|  | EF2-R | CCTTGTAAACCAGAACCAAA | Schaer et al. 2013 |
| *PAT* (nuclear) | DrugT-OF | CAGGDTTRGAYGGATGTGAYGA | Galen et al. 2018 |
|  | DrugT-OR | GGBACTCTMCCAAACATDGCTA | Galen et al. 2018 |
|  | DrugT-IF | CAGTCGGGCGTCATCATRCCWGCAAGTTTYAGAGC | Galen et al. 2018 |
|  | DrugT-IR | GGAAACAGCTATGACCATWGTNCCHGTRAAWCCTTC | Galen et al. 2018 |
|  | CAG* | CAGTCGGGCGTCATCA | Galen et al. 2018 |
|  | M13* | GGAAACAGCTATGACCAT | Galen et al. 2018 |

*cytb*, cytochrome b; *cox1*, cytochrome oxidase I; *clpC*, apicoplast caseinolytic protease; *ef2*, nuclear elongation factor 2; *PAT*, pantothenate transporter. PCR primers were used for sequencing except for *pat*, where the inner primers for the nested PCR incorporated CAG or M13R tags, which were used for subsequent sequencing, following (17). The *cytb* sequences were amplified in two parts with the primers DW2/3932R and 3932F/DW4 (targeting *Plasmodium, Nycteria, Polychromophilus*) or HepCytbF3/HepCytbR3 (targeting *Hepatocystis*). The *cox1* gene was amplified using a nested PCR approach (outer reaction: cox1-out-F/R; nested reaction: cox1-in-R/in-F). The *clpC* and *pat* gene sequences also were amplified using a nested PCR approach (*clpC*: outer reaction: clpC-out-F/R; nested reaction: clpC-in-R/in-F; *pat*: outer reaction: DrugT-OF/-OR; nested reaction: DrugT-IF/-IR). The nuclear *ef2* gene was amplified using the primers EF2-F/R.

**SI References**

Arnuphapprasert, A., Riana, E., Ngamprasertwong, T., Wangthongchaicharoen, M., Soisook, P., et al., 2020. First molecular investigation of haemosporidian parasites in Thai bat species. *Int. J. Parasitol. Parasites Wildl.* 13, 51–61. doi:10.1016/j.ijppaw.2020.07.010

Duval, L., Robert, V., Csorba, G., Hassanin, A., Randrianarivelojosia, M., et al., 2007. Multiple host-switching of haemosporidia parasites in bats. *Malar. J.* 6, 157. doi:10.1186/1475-2875-6-157

Galen, S.C., Borner, J., Martinsen, E.S., Schaer, J., Austin, C.C., et al., 2018. The polyphyly of *Plasmodium*: comprehensive phylogenetic analyses of malaria parasites reveal widespread taxonomic conflict. *R. Soc. Open Sci.* 5, 171780. doi:10.1098/rsos.171780

Table S2. Overview of PCR temperature protocols of the study

| **Gene** | **Primer names** | **Standard/**  **out/nest** | **Initial Denaturation °C, sec** | **No. of cycles** | **Denaturation °C, sec** | **Annealing °C, sec** | **Elongation °C, sec** | **Final Elongation °C, sec** |
| --- | --- | --- | --- | --- | --- | --- | --- | --- |
| *cytb* | DW2/3932R  3932F/DW4  HepCytbF/R | standard | 90°, 240s | 35 | 90°, 60s | 48°, 15s | 68°, 60s | 68°, 600s |
| *cox1* | cox1F/R | out | 94°, 240s | 35 | 94°, 20s | 60°, 30s | 68°, 50s | 68°, 420s |
|  | cox1inF/in-R | nest | 94°, 60s | 40 | 94°, 20s | 52°, 20s | 68°, 50s | 68°, 420s |
| *clpC* | clpC-out-F/R | out | 94°, 240s | 35 | 94°, 20s | 50°, 20s | 68°, 60s | 68°, 420s |
|  | clpC-in-F/R | nest | 94°, 60s | 40 | 94°, 30s | 48°, 30s | 62°, 30s | 68°, 420s |
| *ef2* | Ef2-F/R | standard | 94°, 240s | 40 | 94°, 60s | 52°, 20s | 68°, 60s | 68°, 600s |
| *PAT* | DrugT-OR/OF | out | 94°, 30s | 35 | 94°, 30s | 47°, 30s | 62°, 45s | 62°, 90s |
|  | DrugT-IR/IF | nest | 94°, 30s | 35 | 94°, 30s | 47°, 30s | 62°, 45s | 62°, 90s |

Table S3. Information for Bayesian inference analyses

| **Dataset** | **Alignment length/n sequences** | **Gene seq positions in alignment** | **Missing data** | **Subsets calculated in PartitionFinder vs2** | **Best model (model selection BIC)** | **Run* (n generation)** |
| --- | --- | --- | --- | --- | --- | --- |
| **Concat. dataset (Fig. 2)** | 2,541 nt, no gaps/ n = 76 | Cytb = 1-1110; Cox1 = 1111-2541 | 35.7 % | Subset1 = 1111-2541\3 1-1110\3 | nst=6 rates=invgamma | 10 million, temp=0.02 |
|  |  |  |  | Subset2 = 1112-2541\3 2-1110\3 | nst=6 rates=invgamma |  |
|  |  |  |  | Subset3 = 1113-2541\3 3-1110\3 | nst=6 rates=gamma |  |
| ***Cytb***  **(Fig. 3, Fig. S4)** | 927 nt, no gaps/ n = 124 | Cytb = 1-927 | 3 % | Subset1 = 1-927\3 | nst=6 rates=gamma | 25 million, temp=0.035 |
|  |  |  |  | Subset2 = 2-927\3 | nst=2 rates=invgamma |  |
|  |  |  |  | Subset3 = 3-927\3 | nst=6 rates=invgamma |  |
| ***Cox1***  **(Fig. 4C)** | 915 nt, no gaps/ n = 73 | Cox1 = 1-915 | 0.2 % | Subset1 = 1-915\3 | nst=6 rates=invgamma | 20 million, temp=0.02 |
|  |  |  |  | Subset2 = 2-915\3 | nst=6 rates=invgamma |  |
|  |  |  |  | Subset3 = 3-915\3 | nst=6 rates=gamma |  |
| ***clpC***  **(Fig. 4A)** | 504 nt, no gaps/ n = 73 | clpC = 1-504 | 3.6 % | Subset1 = 1-504\3 2-504\3 | nst=6 rates=invgamma | 30 million, temp=0.02 |
|  |  |  |  | Subset2 = 3-504\3 | nst=6 rates=gamma |  |
| ***PAT***  **(Fig. 4B)** | 831 nt^†^/ n = 53 | Pat = 1-831 | 1.9 % | Subset1 = 1-831\3 2-831\3 | nst=6 rates=invgamma | 10 million, temp=0.02 |
|  |  |  |  | Subset2 = 3-831\3 | nst=6 rates=invgamma |  |

*** Two runs of 4 chains (3 heated, 1 cold) for all runs; except for *cytb* (Fig. 3) and *cox1* (Fig. 4C) where we used 2 runs of 8 chains (7 heated, 1 cold)

^†^ Including gaps

Cytochrome oxidase 1 (*cox1*), cytochrome *b* (*cytb*), apicoplast caseinolytic protease (*clpC*), pantothenate transporter (PF3D7_0206200) (*PAT*)

Table S4. Scale sensitivity. As Table 2, with landscape covariates extracted within a 5 km rather than a 2 km buffer radius around each capture site.

| **Block** | **Predictor** | **Log-odds (89% CrI)** | **SD** | **Marginal effect, pp (89% CrI)** |
| --- | --- | --- | --- | --- |
| Host (individual) | Male vs female | -0.16 (-0.61, 0.26) | 0.27 | -1.9 (-7.9, 3.2) |
| Host (individual) | Reproductive vs non-reproductive | 0.42 (-0.06, 0.92) | 0.31 | 4.8 (-0.7, 11.8) |
| Host (individual) | Body condition (per SD) | -0.12 (-0.37, 0.14) | 0.16 | -1.3 (-4.5, 1.7) |
| Host (species-level trait) | Foliage vs cave roosting | 0.09 (-1.20, 1.39) | 0.81 | 1.0 (-15.9, 16.3) |
| Host (species-level trait) | Roost size <100 vs <10 | -0.71 (-1.97, 0.58) | 0.80 | -7.8 (-25.8, 6.7) |
| Host (species-level trait) | Roost size <1000 vs <10 | 0.50 (-0.83, 1.82) | 0.82 | 7.3 (-11.7, 27.6) |
| Host (species-level trait) | Frugivorous vs insectivorous | 0.02 (-1.38, 1.43) | 0.88 | 0.2 (-16.8, 18.7) |
| Host (species-level trait) | Foraging guild III vs II | 0.29 (-1.03, 1.57) | 0.81 | 3.4 (-12.3, 21.2) |
| Host (species-level trait) | Foraging guild IV vs II | 0.01 (-1.40, 1.43) | 0.87 | 0.2 (-16.9, 17.8) |
| Environment | Antecedent rainfall, 3 mo (per SD) | -0.00 (-0.40, 0.41) | 0.25 | -0.1 (-4.6, 5.6) |
| Environment | Wet season vs dry | -1.19 (-2.08, -0.33) | 0.54 | -13.4 (-26.8, -3.6) |
| Landscape | Agriculture vs forest | -0.40 (-1.35, 0.60) | 0.61 | -4.9 (-18.4, 7.8) |
| Landscape | Mosaic vs forest | -0.52 (-1.35, 0.30) | 0.52 | -6.1 (-18.2, 3.7) |
| Landscape | Edge density (per SD) | -0.28 (-0.76, 0.19) | 0.30 | -3.0 (-8.5, 2.3) |
| Landscape | Human population density (per SD) | -0.62 (-1.18, -0.08) | 0.35 | -6.3 (-12.7, -0.8) |

Table S5. Sampling design balance. Landscape and demographic composition of probabilistically and opportunistically sampled sites in the analyzed sample.

**(a) Site-level landscape covariates**

| **Covariate** | **Probabilistic** | **Opportunistic** |
| --- | --- | --- |
| Edge density (m/ha) | 10.7 [1.7–16.6] | 7.3 [2.0–8.5] |
| Human population density (/km2) | 33 [9–144] | 199 [97–213] |
| Proportion forest | 0.28 [0.00–0.54] | 0.01 [0.00–0.07] |
| Proportion agriculture | 0.23 [0.00–0.88] | 0.87 [0.81–0.94] |
| *Values are median [interquartile range] across sites. Proportions are of modelled focal habitat; the three focal cover proportions sum to one and mosaic is the omitted reference class.* | | |

**(b) Distribution of individuals and sites**

| **Variable** | **Level** | **Probabilistic** | **Opportunistic** |
| --- | --- | --- | --- |
| Total |  | 439 (77) | 150 (7) |
| Host genus | Cynopterus | 311 (56) | 0 (0) |
| Host genus | Hipposideros | 44 (13) | 74 (5) |
| Host genus | Scotophilus | 83 (23) | 0 (0) |
| Host genus | Taphozous | 1 (1) | 76 (6) |
| Roost type | caves | 45 (14) | 150 (7) |
| Roost type | foliage | 394 (73) | 0 (0) |
| Land cover class | ag | 162 (31) | 77 (3) |
| Land cover class | forest | 140 (22) | 49 (1) |
| Land cover class | mosaic | 137 (24) | 24 (3) |
| *Values are number of individuals (number of sites). Site counts do not sum within a variable, as a site may contribute individuals to more than one category. Opportunistic sites were selected to target known roosts and are not a random sample; all opportunistically sampled individuals were cave-roosting bats.* | | | |

Table S6. Prior sensitivity. Population-level coefficients and group-level standard deviations under three prior specifications: the weakly regularizing priors used for the primary analysis, the brms default priors, and an alternative specification with directionally informative priors on body condition, edge density, and human population density.

| **Parameter** | **Regularizing (primary)** | **brms default** | **Original submission** |
| --- | --- | --- | --- |
| Intercept | -1.11 (-2.82, 0.58) | -3.29 (-10.75, 5.55) | -1.12 (-2.84, 0.64) |
| Male vs female | -0.18 (-0.61, 0.25) | -0.19 (-0.56, 0.13) | -0.19 (-0.61, 0.24) |
| Reproductive vs non-reproductive | 0.44 (-0.04, 0.94) | 0.48 (-0.00, 1.04) | 0.44 (-0.05, 0.93) |
| Body condition (per SD) | -0.12 (-0.38, 0.14) | -0.13 (-0.35, 0.07) | -0.12 (-0.37, 0.12) |
| Foliage vs cave roosting | 0.09 (-1.18, 1.37) | -0.70 (-6.66, 3.96) | 0.09 (-1.24, 1.39) |
| Roost size <100 vs <10 | -0.66 (-1.94, 0.65) | -0.03 (-6.39, 5.21) | -0.67 (-1.94, 0.65) |
| Roost size <1000 vs <10 | 0.51 (-0.81, 1.83) | 3.40 (-6.64, 14.47) | 0.52 (-0.82, 1.82) |
| Frugivorous vs insectivorous | 0.00 (-1.40, 1.40) | 1903.13 (-6868.94, 8459.44) | -0.00 (-1.39, 1.42) |
| Foraging guild III vs II | 0.33 (-0.95, 1.61) | 0.70 (-5.59, 6.81) | 0.33 (-0.94, 1.58) |
| Foraging guild IV vs II | 0.02 (-1.36, 1.40) | -1900.53 (-8464.88, 6872.44) | 0.03 (-1.36, 1.41) |
| Antecedent rainfall, 3 mo (per SD) | -0.01 (-0.41, 0.41) | 0.05 (-0.27, 0.42) | -0.00 (-0.40, 0.41) |
| Wet season vs dry | -1.32 (-2.18, -0.48) | -2.02 (-3.01, -0.59) | -1.33 (-2.18, -0.49) |
| Agriculture vs forest | -0.47 (-1.37, 0.45) | -0.75 (-2.68, 1.20) | -0.46 (-1.40, 0.46) |
| Mosaic vs forest | -0.35 (-1.16, 0.48) | -0.26 (-1.59, 1.08) | -0.32 (-1.14, 0.50) |
| Edge density (per SD) | -0.36 (-0.79, 0.06) | -0.59 (-1.36, -0.01) | -0.46 (-0.90, -0.03) |
| Human population density (per SD) | -0.56 (-1.00, -0.12) | -0.66 (-0.97, -0.21) | -0.65 (-1.13, -0.18) |
| SD, host species | 1.41 (0.70, 2.28) | 4.10 (1.80, 7.49) | 1.39 (0.68, 2.25) |
| SD, sampling site | 1.36 (0.94, 1.85) | 1.62 (1.06, 2.09) | 1.38 (0.95, 1.86) |
| *Values are posterior median (89% credible interval) on the log-odds scale. Under brms default priors the feeding guild and foraging guild coefficients are not identified and diverge to large equal-and-opposite values; this reflects the aliasing of species-level traits with the host species varying intercept and is the reason regularizing priors were used for the primary analysis.* | | | |

Table S7. Sampler diagnostics. Convergence and sampling behavior for each fitted model. All models were run as four chains of 4,000 iterations with the first 2,000 discarded as warm-up, adapt_delta = 0.99 and maximum tree depth 12.

| **Model** | **Max R-hat** | **Min bulk ESS** | **Min tail ESS** | **Divergent** | **Max treedepth hits** | **Min E-BFMI** | **Pareto-k > 0.7** |
| --- | --- | --- | --- | --- | --- | --- | --- |
| null | 1.002 | 2254 | 3502 | 0 | 0 | 0.755 | 0 |
| indiv | 1.002 | 2097 | 3971 | 0 | 0 | 0.724 | 0 |
| host | 1.003 | 2339 | 3850 | 0 | 0 | 0.740 | 0 |
| env | 1.003 | 2329 | 3817 | 0 | 0 | 0.748 | 0 |
| land | 1.001 | 2410 | 3639 | 0 | 0 | 0.770 | 0 |
| lean | 1.003 | 2442 | 4017 | 0 | 0 | 0.775 | 0 |
| full | 1.002 | 2133 | 3766 | 0 | 0 | 0.726 | 0 |
| design | 1.002 | 2451 | 3985 | 0 | 0 | 0.809 | 0 |
| *ESS, effective sample size; E-BFMI, energy Bayesian fraction of missing information. Observations with a Pareto-k diagnostic above 0.7 were handled by exact refitting with that observation omitted.* | | | | | | | |

Table S8. Model comparison by approximate leave-one-out cross-validation. Differences in expected log predictive density (ELPD) relative to the best-performing model, with standard errors, and predictive metrics for the binary outcome. All models were fitted to an identical complete-case dataset. The comparison of the full model against the null model, which contains only the intercept and the two varying intercepts, indicates the incremental contribution of the ecological covariates.

| **Model** | **ELPD difference** | **SE of difference** | **LOO AUC** | **Brier score** | **Log score** |
| --- | --- | --- | --- | --- | --- |
| env | 0.0 | 0.0 | 0.829 | 0.128 | -0.397 |
| land | -0.8 | 2.2 | 0.830 | 0.128 | -0.398 |
| null | -0.9 | 1.8 | 0.829 | 0.128 | -0.398 |
| lean | -2.4 | 2.2 | 0.833 | 0.129 | -0.401 |
| design | -2.5 | 2.3 | 0.832 | 0.129 | -0.401 |
| full | -3.0 | 2.3 | 0.832 | 0.129 | -0.402 |
| host | -3.4 | 2.6 | 0.830 | 0.129 | -0.403 |
| indiv | -3.4 | 2.6 | 0.832 | 0.129 | -0.403 |
| *Models: null (intercept and varying intercepts only); indiv (individual-level host covariates); host (all host covariates); env (season and antecedent rainfall); land (landscape covariates); lean (reduced trait set); full (all covariates); design (full model plus a term for sampling design). Differences smaller than their standard errors do not distinguish models.* | | | | | |

**Table S9. Integrated model including a term for sampling design.** As Table 2, with sampling design added as a population-level effect.

| **Block** | **Predictor** | **Log-odds (89% CrI)** | **SD** | **Marginal effect, pp (89% CrI)** |
| --- | --- | --- | --- | --- |
| Host (individual) | Male vs female | -0.17 (-0.60, 0.26) | 0.27 | -2.0 (-7.3, 3.0) |
| Host (individual) | Reproductive vs non-reproductive | 0.45 (-0.05, 0.94) | 0.31 | 4.9 (-0.5, 11.4) |
| Host (individual) | Body condition (per SD) | -0.12 (-0.37, 0.12) | 0.16 | -1.3 (-4.3, 1.5) |
| Host (species-level trait) | Foliage vs cave roosting | -0.01 (-1.30, 1.26) | 0.81 | -0.0 (-16.5, 14.4) |
| Host (species-level trait) | Roost size <100 vs <10 | -0.68 (-1.94, 0.62) | 0.80 | -7.2 (-24.2, 6.8) |
| Host (species-level trait) | Roost size <1000 vs <10 | 0.49 (-0.87, 1.83) | 0.84 | 6.6 (-12.1, 26.5) |
| Host (species-level trait) | Frugivorous vs insectivorous | -0.00 (-1.42, 1.42) | 0.88 | -0.1 (-16.3, 17.8) |
| Host (species-level trait) | Foraging guild III vs II | 0.33 (-0.99, 1.61) | 0.81 | 3.7 (-11.0, 21.0) |
| Host (species-level trait) | Foraging guild IV vs II | -0.00 (-1.44, 1.44) | 0.90 | -0.2 (-16.3, 17.1) |
| Environment | Antecedent rainfall, 3 mo (per SD) | -0.01 (-0.40, 0.39) | 0.25 | -0.1 (-4.4, 5.1) |
| Environment | Wet season vs dry | -1.33 (-2.16, -0.50) | 0.52 | -14.5 (-27.8, -5.1) |
| Landscape | Agriculture vs forest | -0.48 (-1.40, 0.44) | 0.58 | -5.4 (-18.1, 5.2) |
| Landscape | Mosaic vs forest | -0.36 (-1.19, 0.47) | 0.51 | -4.2 (-15.4, 5.8) |
| Landscape | Edge density (per SD) | -0.36 (-0.79, 0.07) | 0.27 | -3.6 (-8.3, 0.8) |
| Landscape | Human population density (per SD) | -0.48 (-0.94, -0.02) | 0.29 | -4.8 (-10.1, -0.2) |
| Sampling design | opportunistic vs probabilistic | -0.78 (-1.93, 0.38) | 0.73 | -7.9 (-20.3, 4.5) |
| *Reference level for sampling design is probabilistic. Because all opportunistically sampled individuals were cave-roosting bats, the design term is identified only from contrasts among cave sites.* | | | | |

**Table S10. Overview of *Hepatocystis parasite* records from Asian bats**

| **Bat family** | **Host bat species** | **Country** | **Molecular data^a^** | ***Hepatocystis* morpho-species^b^** | **Reference** |
| --- | --- | --- | --- | --- | --- |
| Craesonycteridae | *Craesonycteris thonglongyai* | Thailand | *cytb* (378nt) | ? | Arnuphapprasert et al. 2020 |
| **Emballonuridae** | ***Taphozous melanopogon*** | **Cambodia** | ***cytb, cox1, clpC, ef2, pat*** | **NA** | **this study** |
| Hipposideridae | *H. armiger* | Taiwan | -- | *H. hipposideri* | Manwell and Kuntz 1966 |
|  |  | Thailand | -- | ? | Arnuphapprasert et al. 2020 |
|  | *H. bicolor* | Thailand | -- | ? | Arnuphapprasert et al. 2020 |
|  | ***H. galeritus*** | Malaysia | -- | *H. bainae* | Mialhe and Landau 1977 |
|  |  | Malaysia | -- | *H. rodhaini* | Landau et al. 1976 |
|  |  | **Cambodia** | ***cytb, clpC, ef2*** | **NA** | **this study** |
|  | ***H.* cf. *grandis*** | **Cambodia** | ***cytb, cox1, clpC, ef2*** | **NA** | **this study** |
|  | *H. larvatus* | Cambodia | *cytb* | ? | Duval et al. 2007 |
|  |  | Thailand | *cytb* (378nt) | ? | Arnuphapprasert et al. 2020 |
|  | *H. lekakuli* | Thailand | -- | **NA** | Arnuphapprasert et al. 2020 |
| Pteropodidae | *Balionycteris maculata* | Malaysia | -- | *H. pteropi* | Eyles et al. 1962 |
|  | ***Cynopterus brachyotis*** | Malaysia | -- | *H. pteropi* | Eyles et al. 1962 |
|  |  | **Cambodia** | ***cytb, cox1, clpC, ef2, pat*** | **NA** | **this study** |
|  |  | Singapore | *cytb, cox1, clpC* | NA | Martinsen et al. 2008 |
|  |  | Malaysia | *cytb,18S* | NA | Cox-Singh et al. unpublished data^c^ |
|  |  | Singapore | *cytb* | NA | Perkins and Schall 2002 |
|  |  | Singapore | *cytb* | NA | Low et al. 2021 |
|  |  | Thailand | *cytb* (378nt) | ? | Arnuphapprasert et al. 2020 |
|  |  | Borneo | -- | *H. pteropi* | Van Peenen et al. 1974, Masbar et al. 1981 |
|  | *Cynopterus horsfieldii* | Malaysia | -- | *H. pteropi* | Eyles et al. 1962 |
|  |  | Malaysia | *cytb,18S* | NA | Cox-Singh et al. unpublished data^c^ |
|  |  | Borneo | -- | *H. pteropi* | Van Peenen et al. 1974, Masbar et al. 1981 |
|  | ***Cynopterus sphinx*** | Thailand | -- | *H. garnhami* | Manwell and Kuntz 1966 |
|  |  | **Cambodia** | ***cytb, cox1, clpC, ef2, pat*** | **NA** | **this study** |
|  | ***Eonycteris spelaea*** | **Cambodia** | ***cytb, cox1, clpC, pat*** | **NA** | **this study** |
|  | flying foxes | Ceylon | -- | *H. pteropi* | Garnham 1966^d^ |
|  | flying foxes | India | -- | *H. pteropi* | Garnham 1966^e^ |
|  | *Pteropus* spp. | Malaysia | -- | *H. pteropi* | Garnham 1966^f^ |
|  | *Megaerops ecaudatus* | Malaysia | -- | *H. pteropi* | Eyles et al. 1962 |
|  | ***Megaerops niphanae*** | **Cambodia** | ***cytb, cox1, clpC, ef2, pat*** | **NA** | **this study** |
|  | *Pteropus medius^g^* | Assam | -- | *H. pteropi* | Mackie 1914, Garnham 1966 |
| Rhinolophidae | *R. pearsonii* | Thailand | *cytb* (378nt) | ? | Arnuphapprasert et al. 2020 |
|  | *R. thomasi* | Thailand | *cytb* (378nt) | ? | Arnuphapprasert et al. 2020 |
|  | *R. malayanus* | Thailand | *cytb* (378nt) | ? | Arnuphapprasert et al. 2020 |

^a^ if not indicated, partial sequence length per gene > 400 nt

^b^ described morphospecies or assigned to a morphospecies if morphological data was available

^c^ Cox-Singh et al., unpublished data (nucleotide sequences in GenBank)

^d^ observed by Manson-Bahr in 1911 and published by Wenyon in 1926 as described in Garnham 1966

^e^ observed by Donovan, as described in Garnham 1966

^f^ Richard Green 1933 and Annual Report of Medical Research, Kuala Lumpur, 1951 as described in Garnham 1966

^g^ described as *Pteropus edwardsii*

**References for Table S10** (in addition to references in the main text)

Eyles DE, Dunn FL, Liat LB. Blood parasites of Malayan bats. Med J Malaya. 1962;17:87-88.

Landau I, Miltgen F, Le Bail O, Yap LF. *Hepatocystis* of Malaysia IV. Description of *Hepatocystis rodhaini* n. sp., parasite of Microchiroptera. Ann Parasitol Hum Comp. 1976;51:303-307. doi:10.1051/parasite/1976513303.

Mackie FP. Note on the parasite of bat malaria. Indian J Med Res. 1914;2(1):375-376.

Manwell RD, Kuntz RE. *Hepatocystis* in Formosan mammals with a description of a new species. J Protozool. 1966;13(4):670-2. doi:10.1111/j.1550-7408.1966.tb01979.x.

Masbar S, Palmieri JR, Marwoto HA, Purnomo, Darwis F. Blood parasites of wild and domestic animals from South Kalimantan (Borneo), Indonesia. Southeast Asian J Trop Med Public Health. 1981;12(1):42-46.

Perkins SL, Schall JJ. A molecular phylogeny of malarial parasites recovered from cytochrome *b* gene sequences. J Parasitol. 2002;88(5):972-8. doi:10.1645/0022-3395(2002)088[0972:AMPOMP]2.0.CO;2.

Van Peenen PF, Joseph SW, Saleh A, Light RH, Sukeri S, See R. The Indonesian developmental area study: observations on mammals from South and East Kalimantan (Borneo). Southeast Asian J Trop Med Public Health. 1974;5(3):390-397.
