# Supplementary material for: High diversity and novel host associations of bat haemosporidian parasites in Cambodia: insights from a nationwide bat survey and multilocus analyses
Source: Parasit Vectors. 2026 Oct 8;19:436. doi: 10.1186/s13071-026-07732-3 (PMC13651332; doi:10.1186/s13071-026-07732-3)
Supplement: Supplementary file 2 [file 13071_2026_7732_MOESM2_ESM.zip › revised_Additional file 2_Dataset S1/README_sequence_alignments.rtf]

README – sequence alignments

Description
This folder contains FASTA-formatted sequence alignments used for the phylogenetic analyses in the manuscript:
“Haemosporidian diversity in Cambodian bats reveals Hepatocystis diversification and cross-order host switching in mammals.”

File contents
1. infile_figure2_Concat_cytb_cox1_Polychromophilus
   Concatenated alignment of cytochrome b sequences and cytochrome oxidase 1 used for Polychromophilus parasite phylogenetic analyses.
   Format: FASTA

2. infile_figure3_cytb_Hepatocystis
   Aligned cytochrome b sequences used for Hepatocystis parasite phylogenetic analyses.
   Format: FASTA

3. infile_figure4A_clpc_Hepatocystis
   Alignment of apicoplast clpc sequences used for Hepatocystis parasite phylogenetic analyses.
   Format: FASTA

4. infile_figure4B_pat_Hepatocystis
   Alignment of nuclear PAT sequences used for Hepatocystis parasite phylogenetic analyses.
   Format: FASTA

5. infile_figure4C_cox1_Hepatocystis
   Alignment of mitochondrial cytochrome oxidase 1 sequences used for Hepatocystis parasite phylogenetic analyses.
   Format: FASTA


Alignment notes
- Sequences are provided as aligned FASTA files.
- Indel gaps introduced during alignment are indicated by “-”.
- Missing or ambiguous nucleotides are indicated by standard IUPAC nucleotide codes.
- All alignments are provided in the orientation used for downstream analyses.
- Alignment lengths, number of sequences etc. are summarized in Table S4

Use
These alignments were used for phylogenetic analyses in the main manuscript and supplemental files.

Citation
If using these files, please cite the associated publication:
[full citation once available]

Contact
Juliane Schaer
Humboldt University Berlin
[schaerju@hu-berlin.de]
